# Two‐Dimensional Layered Materials for Aqueous Zinc‐Ion Batteries: Multifunctional Roles and Mechanistic Insights

**DOI:** 10.1002/advs.76475

**Published:** 2026-07-28

**Authors:** Shuge Dai, Chenke Yang, Yunrui Jiang, Gaopan Liu, Jianbin Zhou, Ye Wang, Longhui Zeng, Yuen Hong Tsang

**Affiliations:** ^1^ Key Laboratory of Material Physics of Ministry of Education, and School of Physics Zhengzhou University Zhengzhou People's Republic of China; ^2^ Department of Electrical and Computer Engineering University of California San Diego USA; ^3^ University of Science and Technology of China Department of Applied Chemistry Hefei National Laboratory for Physical Sciences at the Microscale Hefei People's Republic of China; ^4^ Department of Applied Physics Photonics Research Institute Research Institute for Advanced Manufacturing The Hong Kong Polytechnic University Hong Kong People's Republic of China; ^5^ Shenzhen Research Institute The Hong Kong Polytechnic University Shenzhen People's Republic of China

**Keywords:** hydrogen evolution reaction, multifunctional applications, two‐dimensional layered materials, zinc dendrites, zinc‐ion batteries

## Abstract

The critical need for safe, scalable grid‐scale energy storage has positioned aqueous zinc‐ion batteries (AZIBs) as a leading solution. However, their commercial deployment faces significant challenges: dendrite growth and corrosion at the anode, and dissolution and slow reaction kinetics at the cathode. Two‐dimensional (2D) layered materials, with their unique structural and chemical properties, are emerging as pivotal enablers to overcome these hurdles. This review systematically analyzes the multifunctional applications of 2D layered materials (graphene derivatives, MXenes, TMDs, etc.) as protective anode layers, high‐capacity cathode hosts, advanced separators, and electrolyte additives in AZIBs. We provide a mechanistic understanding of how 2D layered materials inhibit zinc dendrite formation, suppress the hydrogen evolution reaction (HER) and other side reactions, enhance cathode stability, and facilitate efficient ion transport. Finally, this review critically examines the key challenges and future research directions for implementing 2D layered materials in high‐performance AZIBs, providing new insights to guide their scalable production and commercial deployment.

## Graphical abstract

This overview summarizes versatile 2D layered materials for AZIB anodes, cathodes, separators and electrolytes, illustrating their dendrite/side reaction suppression mechanisms, and analyzes existing hurdles to propose scalable commercial research outlooks.

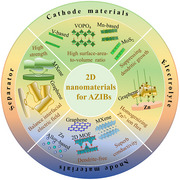

## 1 Introduction

Aqueous zinc‐ion batteries (AZIBs) have garnered significant research and industrial interest as a highly promising technology for grid‐scale energy storage due to a combination of compelling advantages [1, 2, 3, 4, 5]. Their foremost merit lies in intrinsic safety, which stems from the use of non‐flammable aqueous electrolytes, effectively mitigating the risks of fire and explosion associated with organic electrolytes in lithium‐ion batteries [6, 7, 8, 9, 10]. A typical AZIB comprises four fundamental components: a zinc‐based anode, a cathode, a separator, and the electrolyte [11, 12]. The anode is typically made of metallic zinc, which boasts a high theoretical gravimetric capacity (820 mAh g^−1^), low redox potential (−0.76 V vs. SHE), and favorable stability in water [13, 14, 15]. Moreover, AZIBs are also more environmentally benign, leveraging abundant zinc, low toxicity, and an aqueous chemistry that allows for straightforward recycling, thus presenting a greener alternative [16, 17]. These attributes collectively position AZIBs as a viable candidate to meet the growing demands for safe, economical, and large‐scale energy storage solutions.

**FIGURE 1:**
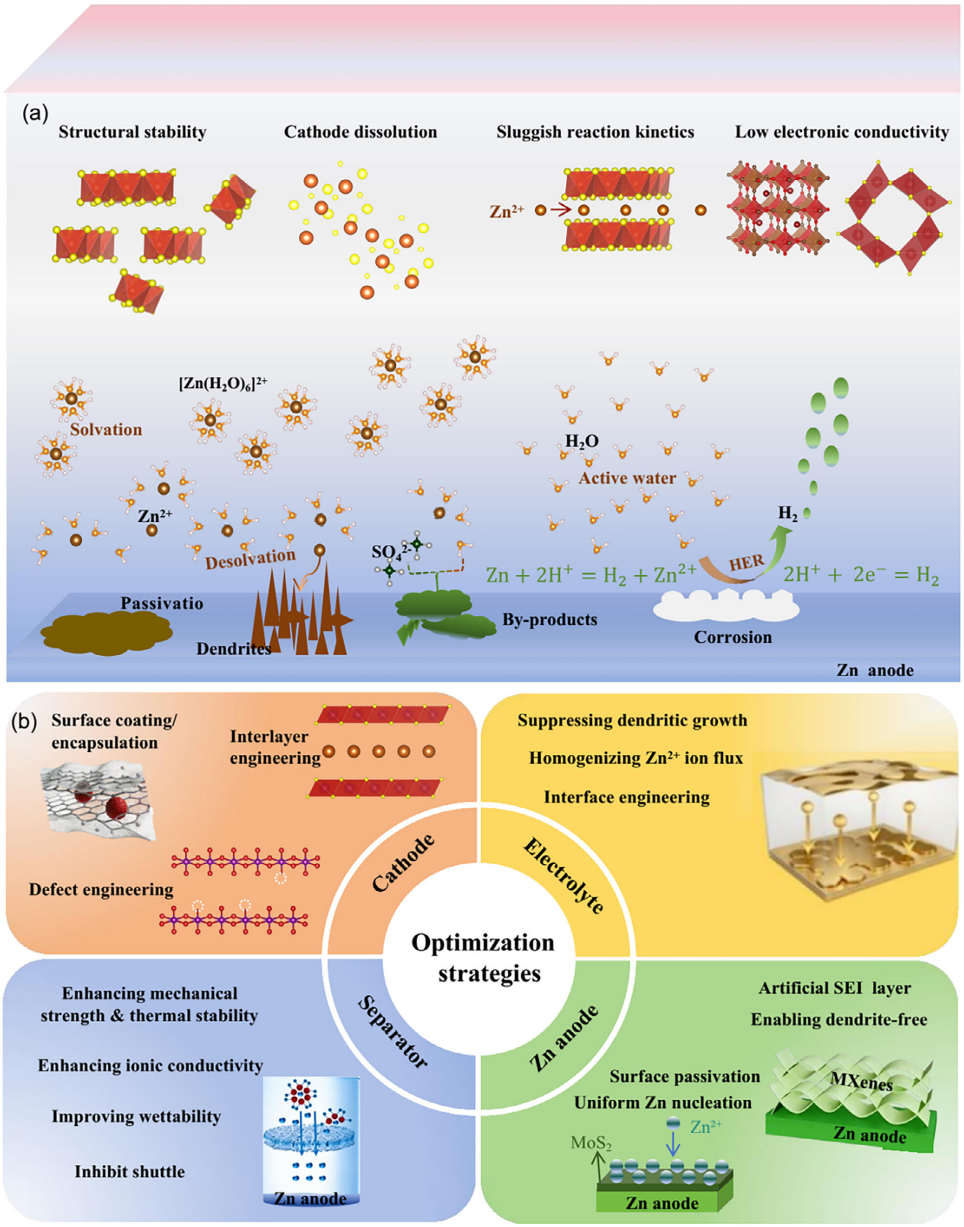
Schematic illustration of (a) the key challenges and (b) optimization strategies for AZIBs.

Despite their considerable promise, the practical deployment of AZIBs is impeded by several fundamental challenges across key battery components, as illustrated in Figure 1a. These issues are interrelated and must be comprehensively addressed to achieve long‐term cycling stability and high efficiency. Notably, the metallic zinc anode suffers from multiple detrimental processes during cycling. For example, the formation of needle‐like zinc protrusions can pierce the separator, leading to internal short circuits and battery failure [18, 19, 20]. Concurrently, the parasitic hydrogen evolution reaction (HER) occurs in acidic or neutral electrolytes, consuming protons and water, which increases local pH, causes electrolyte consumption, and reduces coulombic efficiency [21, 22, 23]. Furthermore, the aqueous environment promotes corrosion of the zinc metal, resulting in uncontrolled self‐discharge and capacity loss. The accumulation of byproducts from these side reactions, such as zinc oxide or hydroxide, forms an insulating passivation layer on the anode surface [24, 25, 26]. This layer impedes Zn^2+^ ion transport, increases interfacial resistance, and exacerbates uneven zinc deposition, further accelerating dendrite formation [27, 28, 29, 30]. Beyond these intrinsic limitations, complex interfacial reactions occur at both electrodes. The solid‐electrolyte interphase (SEI) in AZIBs is often unstable or nonexistent, failing to prevent continuous side reactions between the electrode and the electrolyte, which further accelerates capacity decay and efficiency loss [31, 32, 33, 34, 35]. Consequently, a major focus of current research lies in the strategic modification of key components within aqueous ZIBs, including the cathode, anode, electrolyte, current collector, and separator, to systematically address existing limitations and unlock their full potential for high‐performance, grid‐scale energy storage.

Two‐dimensional (2D) layered materials have emerged as a versatile and highly promising class of materials for addressing the multifaceted challenges in AZIBs (as illustrated in Figure 1b. In this review, 2D layered materials are defined in a broad scope, covering not only intrinsic 2D materials such as graphene derivatives, MXenes, and transition metal dichalcogenides (TMDs) but also 2D‐derived architectures and state‐of‐the‐art interfacial engineering strategies. This inclusive definition allows for a systematic discussion of the multifunctional roles of these materials in resolving the key bottlenecks of AZIBs. The versatile material library encompassing graphene and its derivatives, MXenes, TMDs, and layered double hydroxides (LDHs) affords a broad range of tunable structural, physical, and chemical properties [36, 37, 38, 39, 40]. Benefiting from their distinctive functional characteristics, each category of 2D layered materials can be rationally exploited for diverse functional modules of AZIBs.The exceptional properties inherent to 2D layered materials provide compelling advantages for their application in AZIBs, which can be summarized as follows: First, the expansive planar morphology of 2D layered materials confers an exceptionally high surface‐area‐to‐volume ratio [41, 42, 43]. This property effectively reduces local current density during cycling, promotes a uniform electric field distribution, and is critical for homogenizing Zn^2+^ ion flux, thereby suppressing dendritic nucleation [44, 45]. Second, the rich surface chemistry of many 2D layered materials, such as MXenes (terminated with ─O, ─F, or ─OH groups) and functionalized graphene, offers abundant active sites [46, 47, 48, 49, 50]. These surface groups act as preferential nucleation centers, guide Zn^2+^ migration, and improve electrolyte wettability, collectively fostering uniform and dendrite‐free zinc deposition [51, 52]. Third, the exceptional mechanical strength and flexibility of materials like graphene enable them to serve as robust physical barriers [53, 54]. When applied as interfacial modification layers or integrated into separators, they effectively inhibit zinc dendrite penetration, thereby enhancing safety by mitigating short‐circuit risks [55, 56, 57]. Fourth, the tunable interlayer spacing in many 2D layered materials (e.g., expanded graphene or intercalated MXenes) can be precisely engineered via chemical pre‐intercalation or controlled exfoliation [58, 59, 60]. This tailorability creates optimal diffusion pathways for Zn^2+^ ions, facilitating rapid and highly reversible (de)intercalation kinetics in cathode hosts [61, 62]. Moreover, the exceptional electrical conductivity characteristic can significantly enhance electron transport within the cathode composite. This reduces internal impedance, improves charge transfer efficiency, and ultimately boosts rate capability and overall cathode performance [63, 64, 65]. By integrating these intrinsic properties, 2D layered materials offer a comprehensive toolkit for the concurrent optimization of anode stability, cathode capacity, and ionic transport, presenting a compelling pathway toward high‐performance and durable AZIBs.

In previous review works, single‐component modification or single‐property exploration of 2D materials have been introduced [42, 46]. For example, Zheng et al. delivered a review focusing on the utilization of 2D materials in advanced zinc anodes for AZIBs, bridging the research gap in this specific field. Their work systematically summarized the applications of representative 2D materials in zinc anodes and classified relevant strategies into four categories: protective coatings, host matrices, electrolyte additives, and functional separators [19]. In another representative study, Qiu et al. overviewed the latest advances in manganese‑, vanadium‑, and molybdenum‑based layered cathode materials for AZIBs. They further established a multiscale materials engineering framework spanning from macroscale to microscale to alleviate inherent limitations of layered cathodes, including structural degradation, sluggish electronic conductivity, and retarded ion‑diffusion kinetics [28]. However, a comprehensive review examining how the advantageous properties of 2D layered materials, including high specific surface area, rich surface chemistry, excellent mechanical properties, tunable interlayer spacing, and outstanding electrical conductivity, can address critical bottlenecks in AZIBs across all key components (anode, cathode, separator, and electrolyte) remains absent from the literature [56, 57, 58, 59, 60, 61]. This review lies in systematically integrating the intrinsic advantages of 2D layered materials (including graphene and its derivatives, MXenes, TMDs, LDHs, etc.) with the key challenges faced by AZIBs in practical deployment, thereby constructing a comprehensive and targeted optimization framework for AZIBs. This review establishes systematic correlations between the unique physicochemical characteristics of 2D layered materials and the core technical bottlenecks restricting the practical advancement of AZIBs, with a particular focus on unraveling universal structure‐property‐function relationships and generalized design principles for high‐performance battery optimization. From a component‐oriented perspective, the inherent structural superiorities of typical 2D materials are precisely matched to the key modification demands of different battery units. For cathode engineering, the large specific surface area and tunable interlayer spacing inherent to MXenes and TMDs are clarified as core structural advantages, which can fundamentally mitigate cathode dissolution, suppress harmful shuttle effects, and buffer the volume fluctuation induced by Zn intercalation/deintercalation. For anode regulation, graphene‐based films and LDHs integrate excellent mechanical flexibility and favorable zincophilic surface properties, which can effectively homogenize interfacial ion flux, inhibit zinc dendrite proliferation, and achieve highly uniform and stable Zn^2+^ deposition. In terms of separator modification, ultrathin functional 2D coatings act as efficient physical barriers and precise ion sieves. Their abundant surface‐active sites endow separators with improved ion selectivity and elevated Zn transference number, thereby optimizing the internal ion‐transport behavior of batteries. For electrolyte optimization, emerging 2D material additives and colloidal dispersions are discussed to elucidate their unique regulatory mechanisms in modulating electrolyte solvation structures and stabilizing fragile electrode‐electrolyte interfacial microenvironments. Beyond conventional performance summary, this review further highlights the intrinsic enhancement mechanisms of 2D layered materials toward AZIB performance improvement. It critically identifies and analyzes the unresolved practical challenges restricting their industrial application, including scalable fabrication feasibility, long‐term cycling stability, and economic cost efficiency. Furthermore, prospective and targeted research directions are proposed to guide subsequent in‐depth exploration. This work not only bridges the gap in systematic theoretical summary of 2D material modification strategies for AZIBs but also provides reliable theoretical foundations and practical guidelines for the further development and commercialization of high‐performance AZIBs.

## 2 2D Layered Materials at the Zinc Anode

**FIGURE 2:**
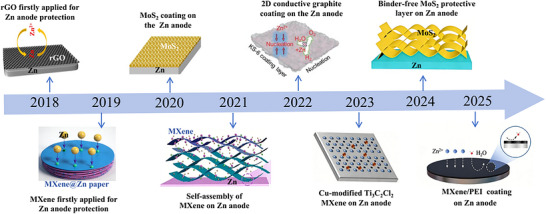
Timeline of the development and application of 2D layered materials for protecting Zn anodes in AZIBs. From left to right: Reproduced with permission [71]. Copyright 2018, American Chemical Society. Reproduced with permission [72]. Copyright 2019, American Chemical Society. Reproduced with permission [73]. Copyright 2020, American Chemical Society. Reproduced with permission [74]. Copyright 2021, Wiley‐VCH GmbH. Reproduced with permission [75]. Copyright 2022, Springer. Reproduced with permission [76]. Copyright 2023, Wiley‐VCH GmbH. Reproduced with permission [77]. Copyright 2024, Elsevier. Reproduced with permission [78]. Copyright 2025, Wiley‐VCH GmbH.

Generally, the unstable zinc anode interface poses a critical challenge that severely limits the cycling life and practical performance of AZIBs. Persistent issues such as uncontrolled dendrite formation, HER, passivation, and corrosion continue to compromise the structural integrity of zinc anodes and reduce Coulombic efficiency, thereby undermining the cycle life and safety of aqueous zinc‐ion batteries [66, 67, 68, 69, 70]. 2D layered materials have emerged as a promising class of modifiers for stabilizing zinc anode interfaces. Their tunable surface chemistry, exceptional mechanical strength, and atomically layered architecture enable uniform zinc deposition and highly reversible plating/stripping processes. Figure 2 illustrates the timeline of the development and application of 2D layered materials for protecting Zn anodes in AZIBs [71, 72, 73, 74, 75, 76, 77, 78].

### 2.1 Persistent Challenges: Dendrites, HER, Passivation, and Corrosion

The uncontrolled deposition of zinc during repeated cycling is a primary cause of dendritic growth, posing a major threat to the stability and safety of zinc anodes [79, 80]. The process initiates during early cycles, where Zn^2+^ ions are reduced at electrochemically favorable charge‐transfer sites, resulting in the nucleation of small zinc protrusions on the anode surface [22]. These protrusions exhibit low surface energy, which promotes the rapid adsorption of free Zn^2+^ ions and facilitates the formation of initial dendritic structures [22, 79]. Furthermore, the high curvature of these nascent dendrites enhances the local electric field, establishing a positive feedback loop that continuously attracts more Zn^2+^ ions and accelerates further growth. Without effective mitigation strategies, these dendrites can ultimately penetrate the separator, resulting in internal short circuits, rapid capacity fade, and catastrophic battery failure [22, 80]. Meanwhile, the aqueous electrolyte environment strongly promotes parasitic HER at the zinc anode interface. This side reaction not only consumes water and produces hydrogen gas but also disrupts ionic equilibrium, causing localized electrolyte concentration gradients and a rise in pH near the electrode surface [79, 80, 81]. The resulting alkaline conditions facilitate the formation of inert by‐products such as zinc hydroxide sulfate or zinc oxide, which deposit on the anode and contribute to surface passivation. This passivation layer markedly reduces the number of active sites available for zinc deposition and hinders ion transport [22]. Besides, the continual generation and accumulation of hydrogen gas increase internal pressure, potentially leading to battery swelling, mechanical deformation, and electrolyte leakage. These phenomena in turn elevate internal resistance, degrade electrical contact, and increase the likelihood of internal short circuits, ultimately impairing the battery's safety, efficiency, and cycle life [82, 83, 84]. Additionally, zinc anodes are highly susceptible to continuous electrochemical corrosion in aqueous electrolytes. This process is driven by the inherent thermodynamic instability of zinc in acidic or neutral media, where it spontaneously reacts with water molecules and dissolved oxygen. The corrosion reaction irreversibly consumes metallic zinc, depleting the active material available for plating/stripping and generating insoluble byproducts (e.g., zinc oxide, zinc hydroxide) that deposit on the electrode surface [82, 83, 84]. These byproducts form a resistive layer that significantly increases interfacial impedance, hindering Zn^2+^ ion transport and charge transfer. As a result, the anode experiences elevated polarization, non‐uniform current distribution, and accelerated capacity fade. Over repeated cycles, the cumulative loss of active zinc and the growing passivation layer lead to premature anode degradation, markedly reducing the cycle life and practical energy density of the AZIBs.

### 2.2 Design Strategies for Anode Protection

The protection of the zinc anode is crucial for enhancing the performance and longevity of AZIBs. Effective design strategies focus on regulating zinc deposition, inhibiting side reactions, and maintaining interfacial stability. Key approaches include the construction of artificial SEI layers and functionalized 3D structures [85, 86, 87, 88, 89, 90, 91, 92]. These strategies aim to achieve uniform ion flux, reduce local current density, suppress dendrite growth, and mitigate corrosion and hydrogen evolution. The following sections detail prominent anode protection designs that contribute to highly reversible and stable zinc anodes.

**FIGURE 3:**
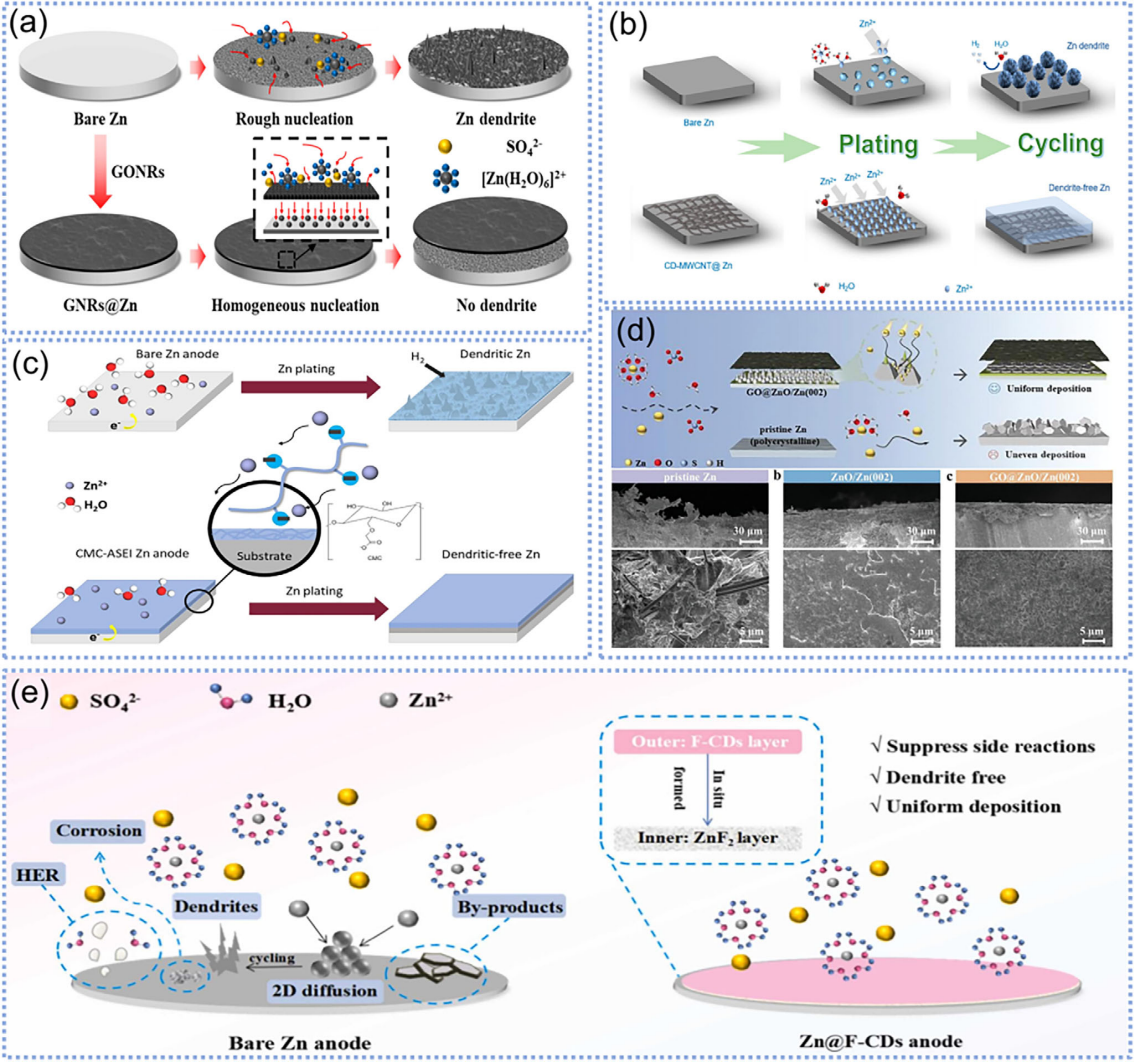
(a) Schematic of the morphological evolution of bare Zn and GNRs@Zn electrodes during stripping/plating cycling [98]. Copyright 2023, Elsevier. (b) Schematic of the dendrite and side reaction suppression mechanism by the CD‐MWCNT coating [99]. Copyright 2024, American Chemical Society. (c) Schematic of the in situ Zn plating/stripping processes for bare Zn vs. the CMC‐SEI Zn composite in aqueous electrolyte [100]. Copyright 2023, Elsevier. (d) Schematic illustration of the plating process on pristine Zn and GO@ZnO/Zn(002) substrates, with corresponding cross‐sectional and top‐view SEM images [101]. Copyright 2024, Wiley‐VCH GmbH. (e) Schematic of Zn deposition on bare Zn vs. Zn@F‐CDs anodes [102]. Copyright 2024, Elsevier.

Artificial SEI layers: Artificially constructed SEI layers based on ultrathin, mechanically robust two‐dimensional (2D) materials (e.g., hexagonal boron nitride and MXene) have emerged as an effective strategy for stabilizing the zinc anode interface [93, 94, 95, 96, 97]. These engineered interphases function as multifunctional protective barriers that physically isolate the anode from direct contact with the aqueous electrolyte, thereby significantly inhibiting water‐induced side reactions. Simultaneously, their high electronic insulation property blocks unwanted electron leakage, while maintained ionic permeability allows regulated Zn^2+^ transport [95, 96, 97]. This synergy effectively suppresses dendrite formation, reduces hydrogen evolution, and mitigates corrosion, contributing to markedly improved interfacial stability and cycling performance in AZIBs. The artificial SEI layer strategy can be summarized into four core design principles: physical isolation with selective ion transport, interfacial homogenization regulation, zincophilic site engineering, and hydrophobic functionality integration. Physical isolation coupled with selective ion transport constitutes the fundamental functionality of artificial SEI layers, requiring the construction of an ultrathin yet mechanically robust protective layer that physically separates the zinc anode from the corrosive aqueous electrolyte while maintaining high ionic permeability and electronic insulation. For example, Wang et al. developed a novel functional interface layer based on graphene nanoribbons (GNRs), which was fabricated by the redox reaction between graphene oxide nanoribbons (GONRs) and a zinc foil (Figure 3a) [98]. The resulting in situ‐formed GNR interlayer acts as an efficient ion‐buffering interface that guides uniform Zn^2+^ nucleation and deposition. As a result, it effectively suppresses the formation of passivation by‐products, mitigates hydrogen evolution reactions, and inhibits severe dendrite growth. SEM images of the cycled electrodes reveal that typical hexagonal zinc dendrites with a size of approximately 15 µm formed on the surface of the pristine zinc foil, exhibiting severe and chaotic aggregation. In contrast, the deposits on the GNRs@Zn surface are smaller than 1 µm and highly uniform, with no discernible dendritic structures. Zincophilic site engineering introduces functional groups or active sites that exhibit high affinity for zinc, substantially lowering the interfacial charge‐transfer energy barrier and guiding uniform Zn^2+^ nucleation and deposition. Kang et al. proposed a β‐cyclodextrin‐modified multiwalled carbon nanotube (CD‐MWCNT) layer as a protective coating for Zn metal anodes (Figure 3b) [99]. The fabricated CD‐MWCNT layer exhibits high affinity toward Zn and significantly reduces the Zn^2+^ transfer energy barrier at the electrode/electrolyte interface (Figure 3c) [100]. This facilitates uniform Zn deposition and effectively suppresses water‐induced side reactions. Hydrophobic functionality integration suppresses hydrogen evolution and corrosion at the anode surface by fundamentally reducing water activity. Tangthuam et al. constructed an artificial SEI based on carboxymethyl cellulose (CMC) on both Zn foil and a Zn‐graphite composite anode [100]. The carboxyl groups within the CMC matrix regulate Zn^2+^ flux and local concentration at the interface, promoting uniform Zn dissolution/deposition. Additionally, these functional groups mitigate corrosion by reducing water activity at the anode surface. Contact angle measurements show that the CMC coating alters the chemical nature of the electrode surface, rendering it more hydrophilic. This promotes uniform electrolyte spreading and prevents localized concentration polarization‐induced pitting and corrosion. Interfacial homogenization regulation emphasizes modulating the interfacial electric field distribution and ion flux through the intrinsic properties of the material. Ye et al. proposed a hybrid strategy that combines crystal reconstruction of commercial zinc foil with a GO protective layer to fabricate a GO@ZnO/Zn(002) anode (Figure 3d) [101]. The GO coating facilitates homogeneous Zn^2+^ flux regulation over a broad area, whereas the dense and uniform ZnO/Zn (002) nanoneedles/nanoparticles strengthen the localized polarized electric field. This synergy promotes rapid Zn^2+^ migration and directs preferential deposition along the (002) crystal plane. Finite‐difference time‐domain (FDTD) simulations confirm that pristine polycrystalline Zn surfaces exhibit severely inhomogeneous electric field distribution. Intense local electric fields concentrate at surface sharp tips, which substantially trigger preferential Zn nucleation and subsequent dendrite propagation. By contrast, the uniquely structured nanoneedle surface of the ZnO/Zn(002) anode achieves a uniformly enhanced local electric field, which efficiently directs Zn deposition toward the base of nanoneedles/nanoparticles and thereby restrains tip‐dominated dendrite growth. Further simulation results of the GO@ZnO/Zn(002) anode reveal that the introduced GO protective layer establishes a long‐range, homogeneous electric field across the entire electrolyte‐anode interface. Such optimized electric field distribution is critical for homogenizing the initial Zn flux and regulating interfacial ion transport behavior. Consequently, this hierarchical design endows the anode with outstanding stability in symmetric cells for 5700 h at 2 mA cm^−2^ and 4200 h at 4 mA cm^−2^, positioning it among the top‐performing Zn anodes in the field [101]. Ge et al. developed a hydrophobic and zincophilic multifunctional F‐CDs protective layer on a pristine Zn anode (Figure 3e) [102]. This F‐CDs layer not only promotes preferential Zn deposition along the (002) plane but also suppresses hydrogen evolution and corrosion of Zn. Therefore, the Zn@F‐CDs anode delivers not only an exceptional cycling stability of over 3500 h at 1 mA cm^−2^ but also a high average Coulombic efficiency of 99.32% for more than 1100 h in half‐cell tests [102]. Overall, the strategic design of artificial SEI layers represents a cornerstone for advancing Zn metal anodes. By integrating materials with tailored properties, these engineered interphases effectively decouple the anode from the corrosive electrolyte. This multi‐pronged protection concurrently addresses the intertwined challenges of dendrites, hydrogen evolution, and corrosion.

**FIGURE 4:**
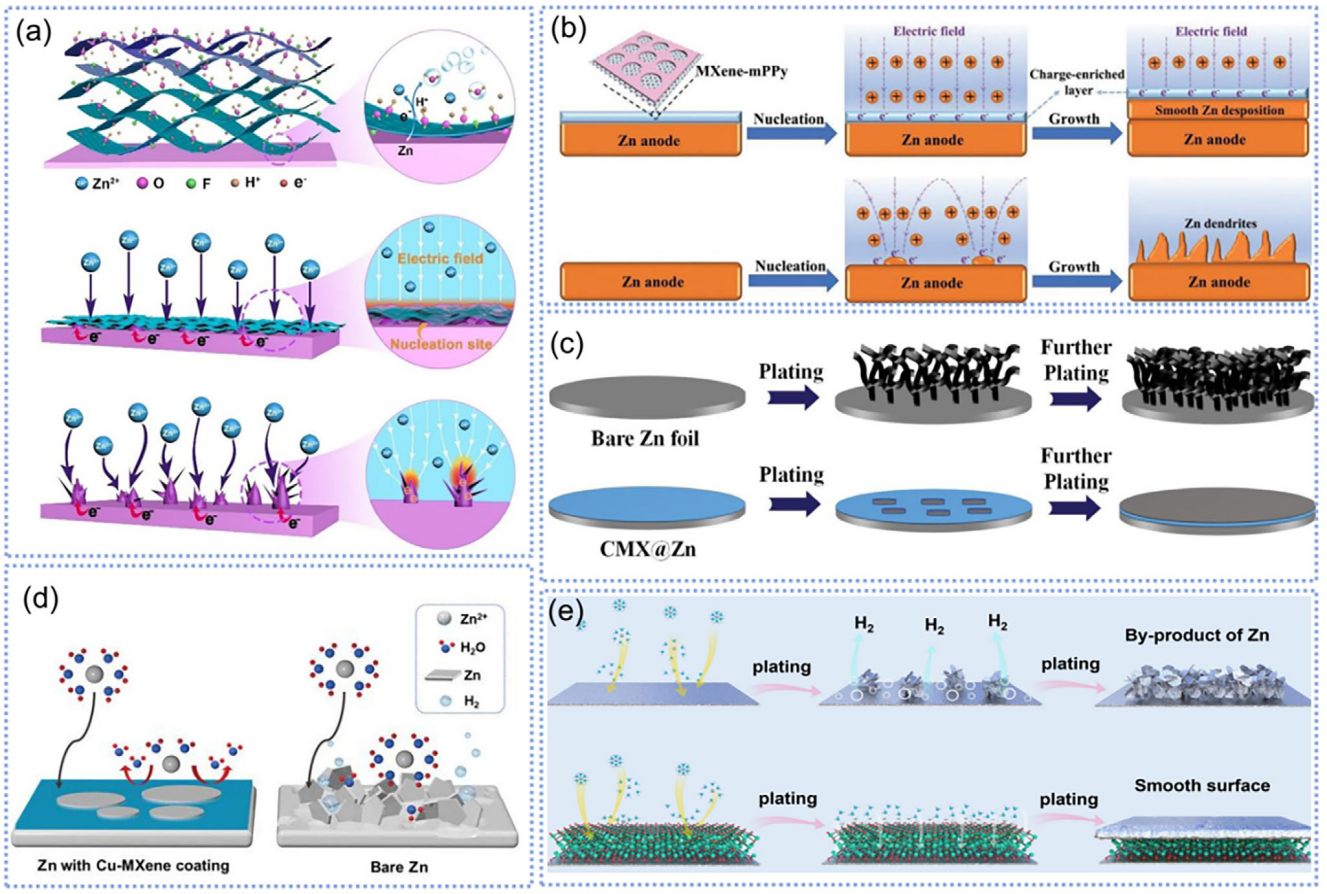
(a) Schematics of the MXene layer formation on Zn foil and the comparative Zn plating behavior on MXene‐coated and pure Zn anodes [80]. Copyright 2021, Wiley‐VCH GmbH. (b) Comparative schematic illustrations of the Zn plating process on MXene‐mPPy/Zn and bare Zn anodes [101]. Copyright 2022, Wiley‐VCH GmbH. (c) Schematic of Zn deposition behavior on bare Zn and CMX@Zn anodes [108]. Copyright 2024, Elsevier. (d) Mechanism schematic of the Cu‐MXene hydrophobic coating for suppressing side reactions on Zn, compared to bare Zn [82]. Copyright 2023, Wiley‐VCH GmbH. (e) Schematic of the protective mechanism of the MXene/PEI coating on zinc [84]. Copyright 2025, Wiley‐VCH GmbH.

Recent advances in artificial interphase engineering highlight MXene‐based coatings as a highly promising approach to stabilizing zinc metal anodes [103, 104, 105, 106]. These functional layers play a critical role in modulating interfacial ion transport, electric field distribution, and nucleation behavior, thereby addressing key challenges such as dendrite growth and water‐induced side reactions. For example, Zhang et al. developed an in situ spontaneously reducing and assembling strategy to construct an ultrathin, uniform MXene layer on the surface of zinc anodes (Figure 4a) [100]. Compared to bare Zn, the MXene coating reduces the Zn nucleation energy barrier and homogenizes the electric field distribution via favorable charge redistribution. As a result, the MXene‐integrated Zn anode exhibits significantly reduced voltage hysteresis and superior cycling stability with dendrite‐free behavior, ensuring high capacity retention and low polarization in zinc‐ion batteries [80]. Zhang et al. proposed a charge‐enrichment strategy to construct dendrite‐free Zn metal anodes through spray‐deposition of MXene‐polypyrrole (MXene‐mPPy) layers with high charge‐storage capability on zinc foil (Figure 4b) [107]. Serving as an artificial interface, the MXene‐mPPy layer enhances charge accumulation and homogenizes the electric field and Zn^2+^ flux distribution at the anode interface. Consequently, the MXene‐mPPy/Zn symmetric cell demonstrates an ultralong cycling stability of over 2500 h, exceptional rate capability, and dendrite‐free Zn deposition morphology.

Shen et al. constructed a CMX‐based protective layer on the Zn surface using a straightforward spin‐coating technique (Figure 4c) [108]. The coating provides abundant nucleation sites and homogenizes the interfacial charge distribution through the zincophilic Ti_3_C_2_Cl_2_ MXene matrix, thereby facilitating uniform Zn deposition. Density functional theory (DFT) calculations demonstrate that the CMX substrate exhibits a higher adsorption energy of Zn atoms on the (002) crystal plane (−0.51 eV) relative to the (100) plane (−0.74 eV). This difference indicates the thermodynamic preference for Zn deposition along the more stable (002) crystal orientation. Furthermore, the CMX substrate delivers a much stronger binding energy with Zn atoms (−2.23 eV) compared with ZMX (‐1.49 eV) and pristine Zn foil (−0.61 eV). The enhanced interfacial binding interaction substantially reduces the Zn nucleation barrier and ultimately facilitates homogeneous Zn nucleation and deposition behavior. Li et al. developed a zincophilic and hydrophobic Cu‐modified Ti_3_C_2_Cl_2_ MXene (denoted as Cu‐MXene) for use as a protective coating on Zn anodes (Figure 4d) [82]. The Cu‐MXene offers abundant nucleation sites and homogenizes the charge distribution, thereby promoting uniform Zn deposition. Furthermore, the hydrophobic coating effectively shields the Zn anode from direct contact with the aqueous electrolyte, helping to suppress side reactions such as hydrogen evolution and corrosion. Consequently, the Cu‐MXene‐coated Zn anode achieves an extended cycling life exceeding 1000 h at 10 mA cm^−2^ with a low polarization below 120 mV, while maintaining a high Coulombic efficiency of over 99.6% for 1100 cycles, demonstrating exceptional electrochemical stability and reversibility [82]. Chen et al. proposed a facile yet robust strategy for modifying Zn anodes through a composite coating of Ti_3_C_2_T_x_ MXene and PEI (Figure 4e) [84]. This MXene/PEI composite incorporates polar functional groups that not only guide uniform zinc deposition but also serve as a barrier to shield the Zn surface from direct contact with active water molecules. Consequently, the modified MXene/PEI@Zn anode exhibits exceptional cycling stability, achieving over 2600 h at 1 mA cm^−2^ [84]. In brief, MXene‐based artificial interphases have proven to be a versatile and effective strategy for stabilizing Zn metal anodes. Key mechanisms include homogenizing the electric field, regulating Zn^2+^ flux, providing abundant zincophilic sites for uniform nucleation, and in some cases, incorporating hydrophobic units to minimize side reactions. Through rational structural and chemical design, these coatings collectively address the intertwined issues of dendrite formation, hydrogen evolution, and corrosion, contributing to significantly enhanced cycling stability and rate performance.

**FIGURE 5:**
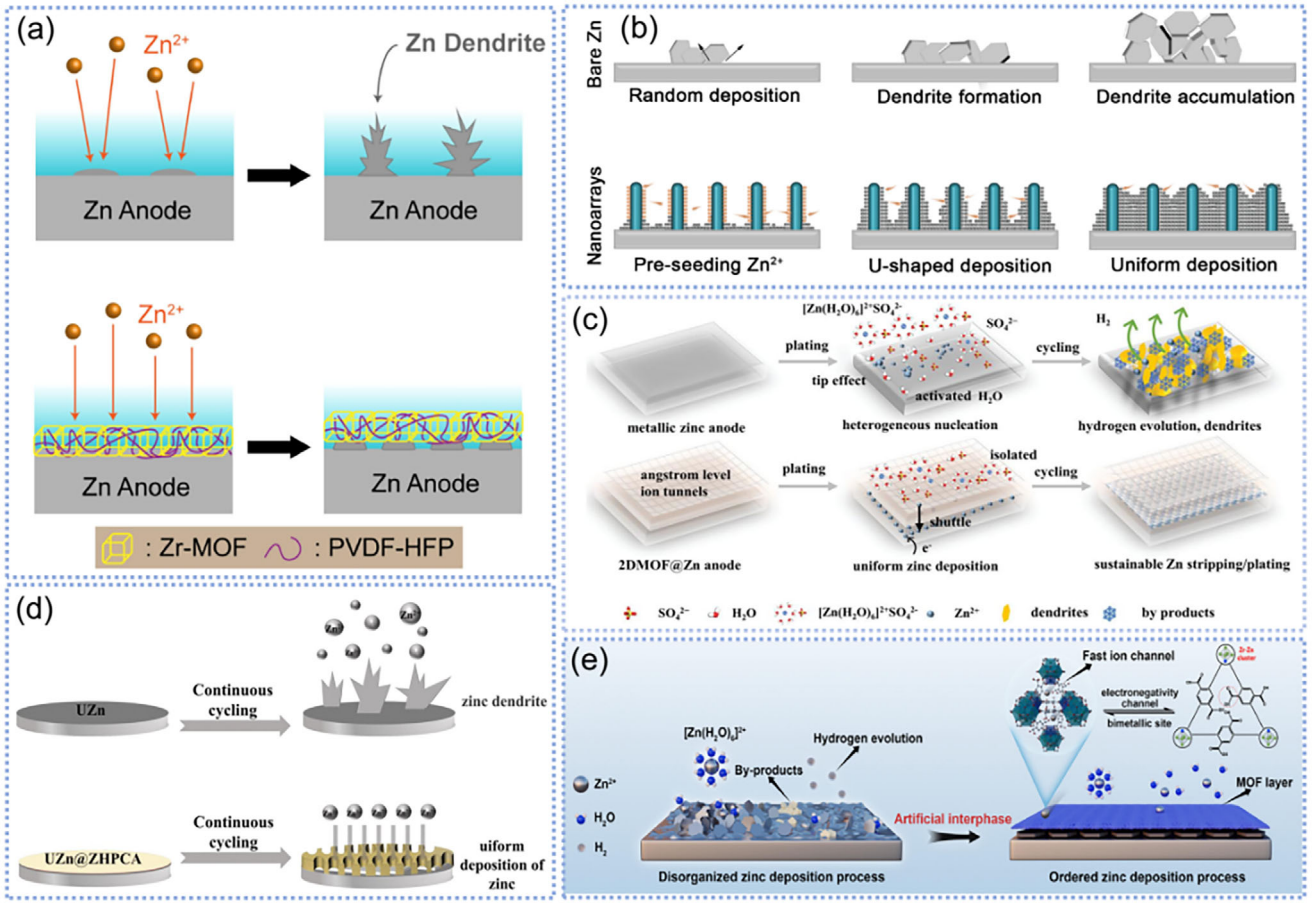
(a) Schematic of dendrite formation on bare Zn vs. the dendrite suppression mechanism by a Zr‐MOF/PVDF‐HFP coating [114]. Copyright 2022, Elsevier. (b) Schematic illustration of guided Zn deposition on a zincophilic MOF nanoarray‐coated Zn substrate vs. unregulated deposition on bare Zn [115]. Copyright 2022, Elsevier. (c) Schematic illustrating the deposition mechanism of Zn on a bare Zn surface vs. a 2D Mn‐MOF@Zn surface [116]. Copyright 2023, Wiley‐VCH GmbH. (d) Schematic of zinc deposition on UZn and ZHPCA@UZn [117]. Copyright 2025, the Royal Society of Chemistry. (e) Schematic illustrating the role of an MOF‐based artificial interlayer in suppressing uneven zinc deposition and mitigating side reactions on bare zinc electrodes [118]. Copyright 2025, Elsevier.

MOFs have emerged as a highly tunable platform for constructing advanced artificial interphases on zinc metal anodes [109, 110, 111, 112, 113]. Their well‐defined porosity, structural diversity, and chemically modifiable surfaces enable precise regulation of ion transport and interfacial electrochemistry, effectively addressing the challenges of dendrite growth and water‐induced side reactions. For instance, Kim et al. utilized a composite protective layer (CPL) composed of a Zr‐based metal–organic framework (UiO‐66(Zr)‐(COOH)_2_) and a poly(vinylidene fluoride‐hexafluoropropylene) (PVDF‐HFP) copolymer binder to suppress dendrite growth (Figure 5a) [114]. The CPL composed of UiO‐66(Zr)‐(COOH)_2_ and PVDF‐HFP copolymer binder is considered a 2D‐derived structure. The highly stable and porous MOF within the CPL functioned as a molecular sieve, enabling uniform Zn^2+^ flux and facilitating homogeneous zinc deposition. This mechanism effectively restrained Zn dendrite formation during cycling. Moreover, the PVDF‐HFP copolymer exhibited high ionic conductivity and strong binding affinity with the Zr‐MOF, which collectively reduced the overpotential of the Zn symmetric cell and enhanced its cycling stability [114]. SEM reveals that the Zr‐MOF/PVDF‐HFP CPL enables the modified Zn anode to maintain a flat and intact surface morphology after long‐term cycling. No obvious dendrite formation or surface degradation is observed, visually verifying the excellent capability of the Zr‐MOF/PVDF‐HFP layer to regulate uniform Zn deposition and suppress dendrite proliferation. To address the dendrite issue in Zn anodes, Wang et al. proposed a U‐shaped deposition architecture enabled by a nanoarray of 2D MOF flakes grown on the Zn surface (Figure 5b) [115]. The abundant zincophilic sites on the MOF flakes facilitate uniform pre‐nucleation of Zn^2^^+^ ions, which is followed by lateral deposition of Zn metal along the flakes and bottom‐up plating from the anode surface, leading to a U‐shaped deposition profile. This MOF nanoarray regulates ion concentration and current distribution, thereby enabling this distinctive deposition mode. By integrating pre‐nucleation and bottom‐up growth mechanisms, this strategy effectively eliminates the “tip effect” and suppresses Zn dendrite formation. Cao et al. developed an innovative 2D Mn‐based MOF with uniform angstrom‐scale ion channels, which mitigated zinc dendrite formation and hydrogen evolution reactions (Figure 5c) [116]. The angstrom‐scale ion tunnels in the 2D Mn‐MOF selectively and efficiently block H_2_O molecules and SO_4_^2^^−^ ions from reaching the zinc surface, thereby effectively suppressing side reactions between the Zn anode and the electrolyte. Sun et al. reported a 2D Zn‐MOF constructed from mixed ligands containing nitrogen and oxygen donors, which can effectively suppress hydrogen evolution and corrosion reactions (Figure 5d) [117]. This unique MOF structure not only facilitates the migration of Zn^2+^ ions but also lowers the activation energy, thereby enhancing zinc ion mobility [113]. Li et al. systematically constructed a functionalized Zr‐Zn bimetallic UiO‐66 (UCOOH‐10 Zn) artificial layer on zinc anodes (Figure 5e) [118]. The intrinsic electronegativity of the carboxyl groups in UCOOH‐10 Zn enhances Zn^2+^ trapping and transport through its channels at the anode‐electrolyte interface. The more electronegative Zn sites in the bimetallic framework facilitate charge transfer near zinc clusters and promote Zn^2+^ migration, collectively enabling uniform zinc deposition [118]. DFT calculations demonstrate that the diffusion energy barrier of migrating Zn is reduced by 0.206 eV in Zn‐substituted channel structures compared with that in pristine Zr‐based channels, facilitating efficient Zn transport kinetics. Moreover, the UCOOH‐10 Zn layer suppresses anion‐induced passivation and mitigates interfacial side reactions, thereby significantly improving the electrochemical stability of the zinc anode. As a result, the UCOOH‐10 Zn‐coated anode achieves stable cycling over 4000 h with a low overpotential of 47.9 mV [118]. In short, MOF‐based artificial interphases represent a versatile and powerful strategy for stabilizing zinc metal anodes. The key to their success lies in their multifunctional design, which combines ion‐sieving capabilities to exclude water and anions, zincophilic nanochannels to guide uniform Zn^2+^ flux and nucleation, and tailored chemical functionality to enhance ion transport kinetics and interfacial stability. By concurrently addressing the issues of uncontrolled deposition and water‐induced side reactions, MOF layers significantly improve the Coulombic efficiency and cycling longevity of AZIBs.

As a rising family of 2D materials, LDHs have been widely recognized as ideal modification materials for AZIB anodes by virtue of their distinctive layered architecture, tunable chemical composition, and superior interfacial regulation ability [103, 104, 105, 106, 107, 108]. The unique layered structure of LDHs can act as a stable mechanical scaffold during zinc deposition, offering inherent ion diffusion pathways to accelerate interfacial Zn transport and steer uniform Zn deposition [117, 118, 119]. Moreover, the inherent hydrophobic properties of LDHs effectively isolate water molecules from the zinc anode surface, significantly alleviating anode corrosion and inhibiting parasitic hydrogen evolution reactions. Benefiting from these structural and functional merits, substantial advances have been made in the practical exploration of LDH‐modified zinc anodes. For example, Liu et al. constructed a multifunctional hybrid interface composed of hydrophobic LDHs and hydrophilic GQDs. In this hybrid structure, LDHs primarily serve hydrophobic protection and mechanical support functions, while GQDs promote uniform deposition of zinc ions along the (002) crystal plane by regulating the solvation environment of Zn^2+^ [119]. Contact angle measurements demonstrate that the pure LDH coating increases the surface contact angle to 81.4°, verifying its favorable hydrophobic feature. COMSOL simulations further reveal that pristine zinc surfaces suffer from severe accumulation of Zn flux and current density at surface protrusions, which easily induces uneven deposition. In comparison, the Zn@LDH@GQDs electrode achieves highly uniform distributions of Zn concentration and interfacial electric field, confirming that the elaborately designed hybrid interface can effectively homogenize ion transport flux and redistribute the surface electric field. The synergistic modulation effect between LDH and GQDs substantially optimizes the interfacial electrochemical environment and ultimately improves the comprehensive electrochemical performance of the zinc anode. Niu et al. constructed a zincophilic ZnAl‐LDH artificial interfacial layer on the surface of zinc anodes exhibiting highly oriented (002) crystal planes through electrochemical methods [81]. This artificial interfacial layer fully utilizes the structural advantages of the (002) crystal plane to physically isolate parasitic reactions. Furthermore, the interfacial layer promotes uniform and rapid zinc deposition/stripping processes from a kinetic perspective, achieving a highly reversible and long‐lifespan zinc metal anode. Notably, the ZnAl‐LDH layer exhibits excellent chemical stability. As a robust physical barrier, ZnAl‐LDH effectively prevents direct contact between the electrolyte and the zinc anode, thereby suppressing interfacial side reactions such as HER and corrosion phenomena. Meanwhile, the zincophilic ZnAl‐LDH coating homogenizes the interfacial electric field and Zn^2+^ concentration distribution, guiding zinc ions to achieve uniform and horizontal deposition, effectively inhibiting dendrite growth. DFT adsorption energy calculations show that the adsorption energy of Zn atoms on the ZnAl‐LDH surface (−1.839 eV) is significantly stronger than that on the Zn(002) crystal plane (−0.383 eV), demonstrating the strong zincophilicity of LDH and its ability to effectively capture Zn^2^^+^ ions. Diffusion barrier calculations further indicate that Zn^2^^+^ exhibits a lower diffusion energy barrier on SZ/LDH@Zn, facilitating rapid Zn^2^^+^ migration. Benefiting from the unique layered structure and hydrophobicity, LDHs provide mechanical support, accelerate ion transport, and suppress corrosion and hydrogen evolution.

COFs represent a class of crystalline porous polymers, which are built up from light elemental building blocks and interconnected by robust covalent bonds into periodic ordered networks. As an indispensable member of 2D materials, 2D COFs are characterized by distinctive planar topological configurations [120, 121, 122, 123, 124, 125]. They have emerged as promising functional materials for AZIBs, particularly for addressing the long‐standing challenges of zinc metal anodes, namely dendrite proliferation, hydrogen evolution reaction (HER), and electrochemical corrosion. Depositing COFs or their composites as protective coatings to construct artificial SEI films has become a prevailing strategy for zinc anode protection [122, 123, 124, 125]. Such artificial interfacial layers can be engineered on zinc substrates through in situ or ex situ approaches, which establish physical and chemical isolation between zinc and electrolyte and effectively modulate interfacial electrochemistry. Duan and co‐workers designed and synthesized a 2D ordered mesoporous COF (2D mCOF) with delocalized electronic states and deployed it as an interfacial modifier for zinc anodes [120]. This material greatly accelerates interfacial reaction kinetics. Its hierarchical mesoporous channels overcome the inherent drawbacks of micropore‐dominated conventional COFs. The microporous framework enables precise size sieving of hydrated zinc ions [Zn(H_2_O)_6_]^2^^+^, which realizes stepwise dehydration within confined spaces and guarantees rapid Zn^2+^ migration even under high‐rate operation [120]. Moreover, nitrogen moieties deliver multi‐site synergistic binding toward Zn^2^^+^ through delocalized electron clouds, which circumvents the degradation issue of conventional functional groups [120]. Consequently, highly reversible and uniform zinc deposition/stripping is achieved, and dendrite growth is substantially inhibited.In the exploration of organic–inorganic hybrid systems, Cao et al. anchored spherical zirconia (ZrO_2_) nanoparticles within COF matrices to fabricate ZrCOF hybrid coatings for zinc anodes [122]. Leveraging the high dielectric property of ZrO_2_, the hybrid triggers Maxwell‐Wagner interfacial polarization and homogenizes the electric field across the electrode‐electrolyte interface [122]. This suppresses the tip effect, increases nucleation density, and induces homogeneous zinc deposition. The COF backbone serves as efficient highways for Zn^2^^+^ transport, while imine (C = N) groups with strong zincophilic affinity facilitate ion adsorption and desolvation [122]. Benefiting from intrinsic hydrophobicity, the coating repels free water and suppresses adverse interfacial side reactions. The well‐designed hybrid interface achieves synergy between electric field homogenization and ion regulation, simultaneously restraining dendrite growth and water‐induced side reactions, boosting desolvation kinetics, and improving the overall cycling durability of zinc anodes. Bifunctional design combining hydrophilicity and hydrophobicity has also been adopted to mitigate dendrites and HER. Guan et al. functionalized COF skeletons with hydrophilic anhydride groups and hydrophobic fluorine atoms [123]. The former captures water molecules and promotes desolvation, whereas the latter excludes bulk water and depresses HER, realizing complementary functions via dual active sites [123]. In another work, Zhong et al. integrated zincophilic Py‐DMTP COF with superhydrophobic Nafion to construct composite coatings [124]. The resultant coatings convert zinc deposition from 2D to 3D diffusion behavior and effectively alleviate concentration polarization.While these artificial SEI layers differ in material design, their underlying working mechanisms follow the same principles. First, the dense or superhydrophobic coatings act as physical barriers to separate zinc from electrolyte water, fundamentally suppressing HER and corrosion. Second, zincophilic functional groups coordinate strongly with Zn^2^^+^, weaken ion‐water interactions, and lower the desolvation energy barrier. Third, the periodically arranged nanopores provide uniform ion transport pathways and homogenize interfacial electric fields, guiding dense and planar zinc deposition to eradicate dendrite formation. Lastly, both organic–inorganic hybridization and dual‐functional group design rely on component synergy to tackle multiple interfacial issues simultaneously. With elaborate modulation including hybrid construction, bifunctional modification, pore architecture engineering, and electronic structure regulation, COF‐based artificial SEIs can integrate multiple functions such as physical isolation, chemical catalysis, and ion steering. This integrated strategy comprehensively resolves the three major obstacles of zinc anodes: dendrite growth, corrosion and HER. These research advances not only elevate the rate capability and cycling stability of AZIBs, but also provide solid theoretical foundations and practical technical routes for the development of next‐generation high‐performance aqueous energy storage devices with application prospects.

**FIGURE 6:**
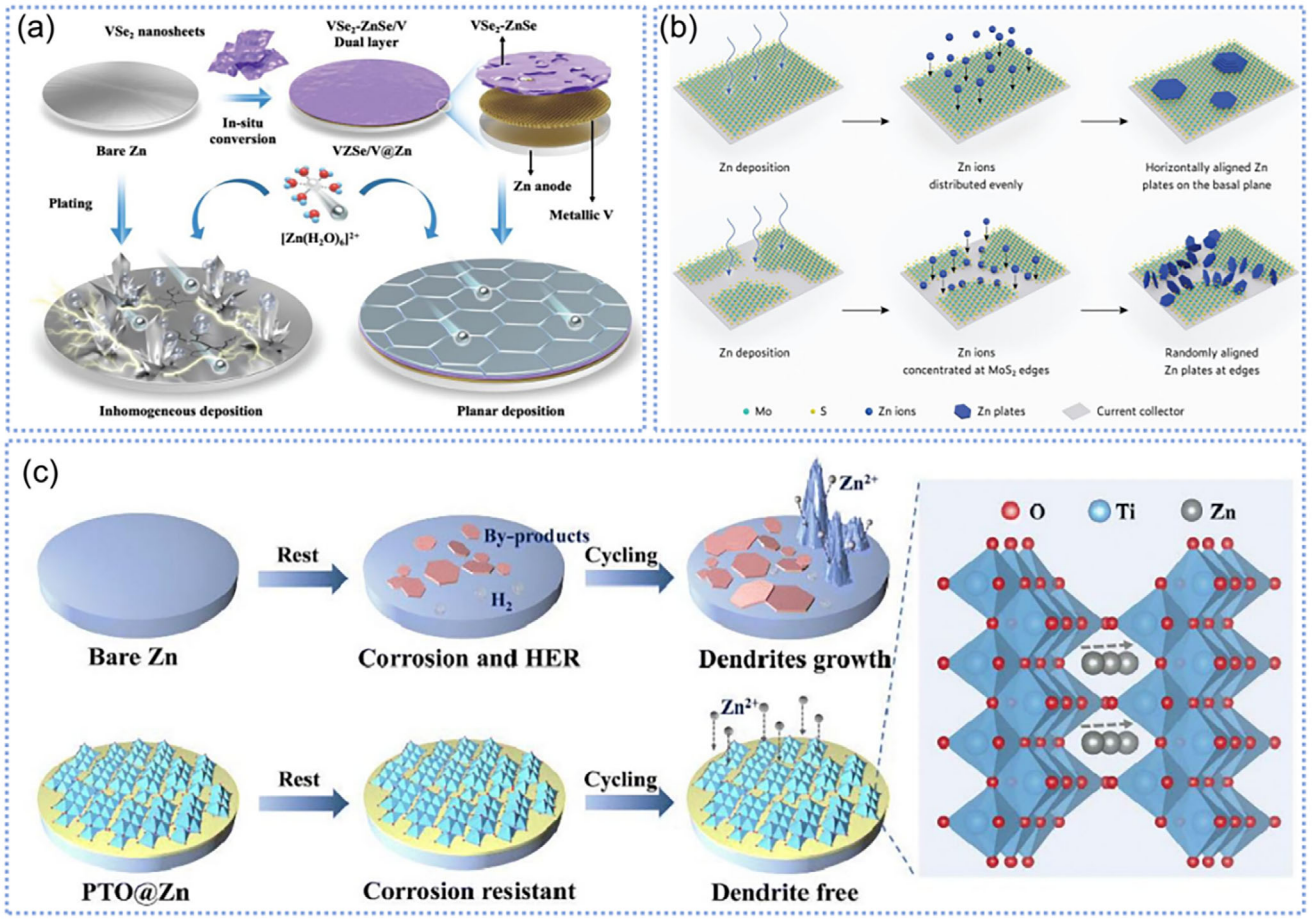
(a) Schematic of the in situ conversion engineering strategy for fabricating a VZSe/V@Zn anode with a dual‐layer structure, composed of a low lattice‐mismatch VSe_2_–ZnSe outer layer and a corrosion‐resistant vanadium inner layer [128]. Copyright 2024, Wiley‐VCH GmbH. (b) Schematic of the competitive reaction pathways for Zn deposition on the basal plane and edge sites of a MoS_2_ substrate [129]. Copyright 2023, Wiley‐VCH GmbH. (c) Schematic illustrations of the electrochemical performance of bare Zn vs. PTO@Zn electrodes [130]. Copyright 2024, the Partner Organisations.

The strategic design of artificial interfacial layers has evolved beyond simple physical barriers to encompass sophisticated structures that actively guide deposition kinetics and interfacial chemistry. Recent studies highlight the efficacy of constructing coupled or composite interfaces with multifunctional components to achieve highly stable zinc metal anodes [119, 120, 121, 122, 123, 124, 125, 126, 127]. A prominent strategy involves creating coupled structures with distinct yet synergistic functions. Zhang et al. developed an in situ conversion strategy to construct a coupled interfacial structure (VZSe/V@Zn) on the zinc anode, enabling long‐term stable and reversible planar zinc deposition (Figure 6a) [128]. The outer hybrid layer composed of VSe_2_ and ZnSe exhibits a low lattice mismatch with the Zn (002) plane, which collectively facilitates ordered planar zinc deposition [128]. Meanwhile, the inner metallic vanadium layer offers excellent corrosion resistance, effectively shielding the anode from electrolyte‐induced degradation. The as‐prepared protective layer enables the zinc anode to achieve an extended cycle life of over 2000 cycles, along with outstanding capacity retention of 93.1% and an ultra‐high average Coulombic efficiency of 100% [128]. Wang et al. developed a strategy for large‐area epitaxial deposition of zinc anodes using mono‐oriented MoS_2_ as a substrate (Figure 6b) [129]. The edges of MoS_2_ exhibit a significantly stronger zinc deposition‐inducing effect compared to the basal plane, thereby strongly suppressing Zn dendrites. Li et al. designed an artificial multifunctional protective layer on zinc foil via a facile doctor‐blading method [126]. This layer consists of 2D Ti_3_O_7_^2−^ nanosheets uniformly dispersed within a polyacrylonitrile (PAN) matrix (Figure 6c) [130]. The PAN matrix serves as an elastic barrier that effectively blocks H_2_O and O_2_, thereby enhancing corrosion resistance and suppressing severe dendrite formation. Table 1 comprehensively summarizes the electrochemical performances of 2D layered materials at the zinc anode. Overall, the advancement of artificial interfaces for zinc anodes is increasingly centered on multifunctional and structurally guided designs. These strategies collectively address the intertwined challenges of dendrite growth and interfacial side reactions by simultaneously regulating the crystallographic, chemical, and mass‐transport properties at the anode‐electrolyte interface. The successful demonstration of long‐cycle‐life anodes underscores the critical importance of rational interface engineering in realizing the full potential of AZIBs.

**TABLE 1:** Summary of the performances of 2D layered materials at the zinc anode.

Electrode material	Electrolyte	Rate performance	Discharge capability	Cycle capability	Ref.
Zn@UIO‐66/rhG_0.1_	2 M ZnSO_4_	49.6% retention (5 A g^−1^)	368.2 mAh g^−1^ (0.1 A g^−1^)	500 cycles, 62.4% retention (1 A g^−1^)	[131]
Zn/rGO@TiO_2_	2 M ZnSO_4_	99.9% retention (0.2 A g^−1^)	162.5 mAh g^−1^ (0.2 A g^−1^)	1000 cycles, 81.3% retention (1 A g^−1^)	[132]
Zn–rGO–Cu	2 M ZnSO_4_	0 retention (5 A g^−1^)	429.9 mAh g^−1^ (0.2 A g^−1^)	900 cycles, 81% retention (0.5 A g^−1^)	[133]
rGO‐0.5‐ZPA	2.5 M Zn(CF_3_SO_3_)_2_	45.5% retention (20 A g^−1^)	323 mAh g^−1^ (0.2 A g^−1^)	2000 cycles, 80% retention (5 A g^−1^)	[134]
aGO‐SA@Zn	2 M ZnSO_4_	—	300 mAh g^−1^ (0.1 A g^−1^)	1500 cycles, 80.6% retention (1 A g^−1^)	[135]
ZGZP	2 M ZnSO_4_	66.6% retention (4 A g^−1^)	120 mAh g^−1^ (0.4 A g^−1^)	1200 cycles, 91.3% retention (2 A g^−1^)	[136]
GO‐CDs	2 M ZnSO_4_	71.9% retention (1.232 A g^−1^)	189 mAh g^−1^ (0.03 A g^−1^)	1700 cycles, 74.6% retention (0.3 A g^−1^)	[137]
Zn@rGO@ZrO_2_	2 M ZnSO_4_	71.4% retention (4 A g^−1^)	105 mAh g^−1^ (0.8 A g^−1^)	1000 cycles, 95.7% retention (2 A g^−1^)	[138]
Zn@LDH@GQDs	2 M Zn(CF_3_SO_3_)_2_	57% retention (10 A g^−1^)	358 mAh g^−1^ (1 A g^−1^)	1000 cycles, 97.9% retention (5 A g^−1^)	[139]
GO/Co‐CuS	3 M Zn(CF_3_SO_3_)_2_	115% retention (1 A g^−1^)	77 mAh g^−1^ (1 A g^−1^)	241 cycles, 72% retention (2 A g^−1^)	[140]
SFG‐Zn	2 M ZnSO_4_ and 0.1 M MnSO_4_	48% retention (2 A g^−1^)	260 mAh g^−1^ (0.3 A g^−1^)	150 cycles, 58% retention (2 A g^−1^)	[141]
h‐WO_3_/3DG	2 M ZnSO_4_	99.8% retention (0.1 A g^−1^)	62.3 mAh g^−1^ (0.1 A g^−1^)	1000 cycles, 76.6% retention (2 A g^−1^)	[142]
Zn_G	2 M ZnSO_4_	59% retention (2 A g^−1^)	220 mAh g^−1^ (0.1 A g^−1^)	1600 cycles, 72.7% retention (0.5 A g^−1^)	[143]
NGO@Zn	2 M ZnSO_4_	70.8% retention (0.74 A g^−1^)	130 mAh g^−1^ (0.03 A g^−1^)	300 cycles, 94% retention (0.15 A g^−1^)	[144]
FLG1 @Zn	2 M ZnSO_4_ and 0.2 M MnSO_4_	56.3% retention (1 A g^−1^)	245.2 mAh g^−1^ (0.1 A g^−1^)	5000 cycles, 96.6% retention (10 A g^−1^)	[145]
MXZA@Zn	2 M ZnSO_4_	35.8% retention (5 A g^−1^)	324.7 mAh g^−1^ (0.1 A g^−1^)	1000 cycles, 98.4% retention (4 A g^−1^)	[146]
ZnF_2_/V_2_CTx@Zn	2 M ZnSO_4_	82.8% retention (10 A g^−1^)	286.9 mAh g^−1^ (0.5 A g^−1^)	1000 cycles, 86.1% retention (5 A g^−1^)	[147]
cZIF@MXene‐Zn	2 M ZnSO_4_	—	400 mAh g^−1^ (0.1 A g^−1^)	4500 cycles, 76.6% retention (4 A g^−1^)	[148]
MVMX@Zn	2 M ZnSO_4_	94% retention (5 A g^−1^)	182.1 mAh g^−1^ (1 A g^−1^)	5000 cycles, 68.5% retention (1 A g^−1^)	[149]
Bi‐MX@ZnP	2 M Zn(CF_3_SO_3_)_2_	51.9% retention (5 A g^−1^)	146.3 mAh g^−1^ (0.2 A g^−1^)	515 cycles, 77.4% retention (0.2 A g^−1^)	[150]
Zn@PT	2 M ZnSO_4_	37.4% retention (5 A g^−1^)	348.2 mAh g^−1^ (0.1 A g^−1^)	1000 cycles, 98.6% retention (5 A g^−1^)	[151]
ZnO@NC/MXene	2 M ZnSO_4_	98.4% retention (1 A g^−1^)	180.5 mAh g^−1^ (1 A g^−1^)	1000 cycles, 71.1% retention (4 A g^−1^)	[152]
PAM@Zn	2 M ZnSO_4_	55.7% retention (1.45 A g^−1^)	320.1 mAh g^−1^ (0.145 A g^−1^)	1500 cycles, 82.4% retention (1.45 A g^−1^)	[153]
VS_2_@MXene	1 M ZnSO_4_	60.5% retention (1 A g^−1^)	207.2 mAh g^−1^ (1 A g^−1^)	5000 cycles, 96% retention (0.1 A g^−1^)	[154]
V_4_C_3_Tx@Zn	2 M ZnSO_4_	63.7% retention (20 A g^−1^)	261.2 mAh g^−1^ (0.2 A g^−1^)	5000 cycles, 74.3% retention (5 A g^−1^)	[155]
MX@Zn	2 M ZnSO_4_ and 0.2 M MnSO_4_	48.6% retention (3 A g^−1^)	—	500 cycles, 64.7% retention (1 A g^−1^)	[156]
MX‐TMA@Zn	2 M ZnSO_4_	49.1% retention (2 A g^−1^)	353.8 mAh g^−1^ (0.2 A g^−1^)	2000 cycles, 99.7% retention (2 A g^−1^)	[157]
ZnGaIn//MXene	2 M ZnSO_4_	67.8% retention (3.2 A g^−1^)	280 mAh g^−1^ (0.2 A g^−1^)	400 cycles, 78.9% retention (3.2 A g^−1^)	[158]
VN/MXene	3 M Zn(CF_3_SO_3_)_2_	29.4% retention (5 A g^−1^)	420 mAh g^−1^ (0.5 A g^−1^)	2000 cycles, 82.8% retention (5 A g^−1^)	[159]
Zn|LM	2 M ZnSO_4_	101% retention (0.2 A g^−1^)	282.7 mAh g^−1^ (0.2 A g^−1^)	1000 cycles, 90% retention (1 A g^−1^)	[160]
NHVO@Ti_3_C_2_Tx	3 M Zn(CF_3_SO_3_)_2_	32.5% retention (1 A g^−1^)	498.4 mAh g^−1^ (0.1 A g^−1^)	6000 cycles, 88% retention (5 A g^−1^)	[161]
MGA@Zn	2 M ZnSO_4_	48.4% retention (0.74 A g^−1^)	126 mAh g^−1^ (0.03 A g^−1^)	600 cycles, 90.3% retention (0.03 A g^−1^)	[162]
MZn‐60	2 M ZnSO_4_	51.8% retention (3 A g^−1^)	281.2 mAh g^−1^ (0.2 A g^−1^)	500 cycles, 81% retention (1 A g^−1^)	[163]
MAF‐4M@Zn	2 M ZnSO_4_	74% retention (4 A g^−1^)	213.3 mAh g^−1^ (0.2 A g^−1^)	300 cycles, 80% retention (2 A g^−1^)	[164]
Zn‐TCPP/Zn	2 M ZnSO_4_	32.9% retention (10 A g^−1^)	340.8 mAh g^−1^ (0.2 A g^−1^)	1000 cycles, 82.5% retention (4 A g^−1^)	[165]
Zn/Mn‐MOF@CNT	1 M Zn(NO_3_)_2_	28.8% retention (1.2 A g^−1^)	459.9 mAh g^−1^ (0.2 A g^−1^)	200 cycles, 93% retention (0.22 A g^−1^)	[166]
ZnCo‐MnO/C	2 M ZnSO_4_ and 0.1 M MnSO_4_	18.9% retention (3 A g^−1^)	428.9 mAh g^−1^ (0.1 A g^−1^)	2000 cycles, 93.8% retention (3 A g^−1^)	[167]
Zn@CPC‐Co	2 M ZnSO_4_ and 0.1 M MnSO_4_	64% retention (2 A g^−1^)	297 mAh g^−1^ (0.1 A g^−1^)	6000 cycles, 46.3% retention (1 A g^−1^)	[168]
ZnO/Zn⊂CF–Zn	2 M ZnSO_4_	33.9% retention (20 A g^−1^)	420.7 mAh g^−1^ (0.2 A g^−1^)	400 cycles, 93% retention (0.5 A g^−1^)	[169]
Zn@CuNOC	2 M ZnSO_4_	58% retention (5 A g^−1^)	272 mAh g^−1^ (0.1 A g^−1^)	400 cycles, 48% retention (0.5 A g^−1^)	[170]
TNGs@Zn	2 M ZnSO_4_ and 0.1 M MnSO_4_	39.6% retention (1 A g^−1^)	331.8 mAh g^−1^ (0.1 A g^−1^)	700 cycles, 57% retention (1 A g^−1^)	[171]
PF@Zn	2 M ZnSO_4_	23% retention (2 A g^−1^)	77.4 mAh g^−1^ (0.5 A g^−1^)	3000 cycles, 96.6% retention (1.5 A g^−1^)	[172]
3D Zn@TB	2 M ZnSO_4_	47.9% retention (5 A g^−1^)	269 mAh g^−1^ (0.25 A g^−1^)	5000 cycles, 97.8% retention (5 A g^−1^)	[173]
Zn@NOG	2 M ZnSO_4_ and 0.4 M MnSO_4_	67% retention (1 A g^−1^)	—	2500 cycles, 99% retention (0.6 A g^−1^)	[174]
ZZCC	2 M ZnSO_4_ and 0.1 M MnSO_4_	50.3% retention (10 A g^−1^)	299.7 mAh g^−1^ (0.5 A g^−1^)	500 cycles, 88.5% retention (2 A g^−1^)	[175]
CFF@Cu	2 M ZnSO_4_	53.3% retention (5 A g^−1^)	255 mAh g^−1^ (0.1 A g^−1^)	500 cycles, 81.3% retention (2 A g^−1^)	[176]
C_flower_/Zn	2 M ZnSO_4_	27.3% retention (5 A g^−1^)	275 mAh g^−1^ (0.1 A g^−1^)	500 cycles, 91% retention (1 A g^−1^)	[177]
Zn@CC‐CNF	2 M ZnSO_4_	—	307 mAh g^−1^ (0.2 A g^−1^)	300 cycles, 49.7% retention (0.1 A g^−1^)	[178]
Zn@ZnO HPA‐2.0	1 M Zn(CF_3_SO_3_)_2_	94.8% retention (0.2 A g^−1^)	230.5 mAh g^−1^ (0.2 A g^−1^)	1000 cycles, 95.7% retention (9 A g^−1^)	[179]
Zn@Cu nanosheets@ACC	1 M ZnSO_4_	—	287 mAh g^−1^ (1 A g^−1^)	1000 cycles, 94.8% retention (1 A g^−1^)	[180]
ZnP/CF	2 M ZnSO_4_	—	210.2 mAh g^−1^ (1 A g^−1^)	7000 cycles, 93.2% retention (1 A g^−1^)	[181]

## 3 2D Layered Materials in Cathode Design

**FIGURE 7:**
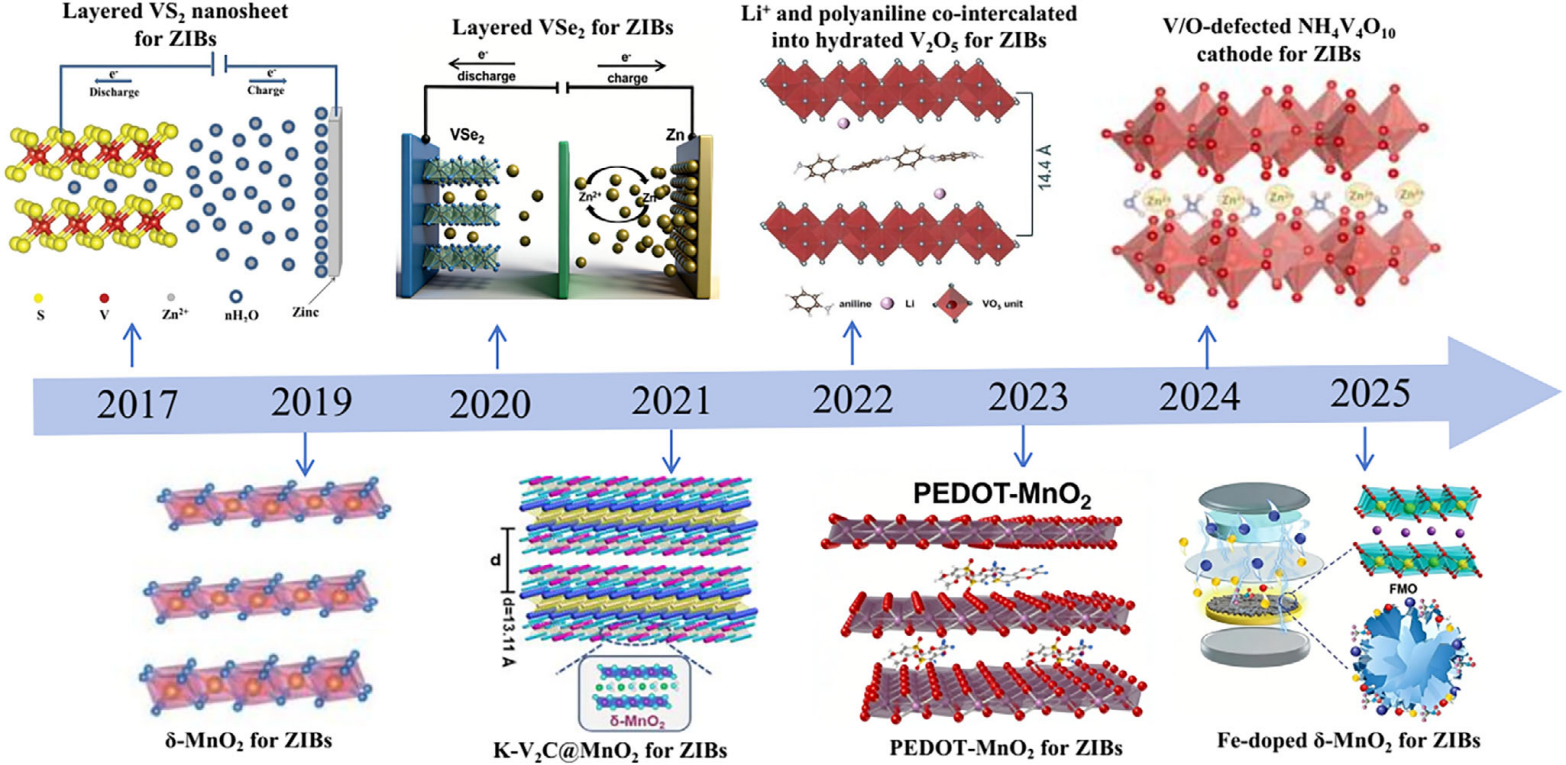
Timeline of the development of 2D layered materials as cathodes in AZIBs. From left to right: Reproduced with permission [191]. Copyright 2017, Wiley‐VCH GmbH. Reproduced with permission [192]. Copyright 2019, Wiley‐VCH GmbH. Reproduced with permission [193]. Copyright 2020, the Royal Society of Chemistry. Reproduced with permission [194]. Copyright 2021, American Chemical Society. Reproduced with permission [195]. Copyright 2022, the Royal Society of Chemistry. Reproduced with permission [196]. Copyright 2023, Elsevier. Reproduced with permission [197]. Copyright 2024, Elsevier. Reproduced with permission [198]. Copyright 2025, Wiley‐VCH GmbH.

2D layered cathode materials play a critical role in determining the energy density, rate capability, and cost of AZIBs. Numerous 2D materials have been explored as cathodes for AZIBs, such as manganese‐based compounds, vanadium‐based composites, transition metal sulfur compounds, and organic materials [182, 183, 184, 185, 186, 187, 188, 189, 190, 191]. Figure 7 illustrates the timeline of the development of 2D layered materials as cathodes in AZIBs [191, 192, 193, 194, 195, 196, 197, 198]. However, their practical application is hindered by several limitations, including low discharge potential, limited storage capacity, sluggish reaction kinetics, and poor structural reversibility during Zn^2+^ insertion/extraction in discharge/charge cycles [199, 200, 201, 202, 203]. A fundamental understanding of the physical and chemical properties of these materials is essential for designing advanced cathodes.

### 3.1 Cathode Material Challenges: Dissolution, Low Conductivity, and Slow Solid‐State Diffusion

The development of high‐performance 2D cathode materials for AZIBs faces several critical challenges that hinder their practical application. First, many cathode materials, particularly manganese‐based oxides and vanadium compounds, are prone to dissolution in aqueous electrolytes [204, 205, 206, 207, 208]. This leads to active material loss, rapid capacity fading, and structural degradation over repeated cycles. Second, limited intrinsic electronic and ionic conductivity in materials such as transition metal sulfides and certain organic frameworks results in high internal resistance, poor rate performance, and inefficient utilization of active material [207, 208, 209, 210]. Third, the strong electrostatic interaction between Zn^2+^ ions and the host lattice creates a high diffusion energy barrier, causing sluggish Zn^2+^ migration kinetics [209, 210, 211, 212]. This limits charge/discharge rates and overall power density. These challenges collectively contribute to suboptimal battery performance, including low specific capacity, reduced cycling stability, and voltage hysteresis. Addressing these issues requires material design strategies such as nanostructuring, composite formation, heteroatom doping, and surface modification to enhance structural stability, electrical conductivity, and ion diffusion pathways.

### 3.2 2D Materials as Active Hosts

**FIGURE 8:**
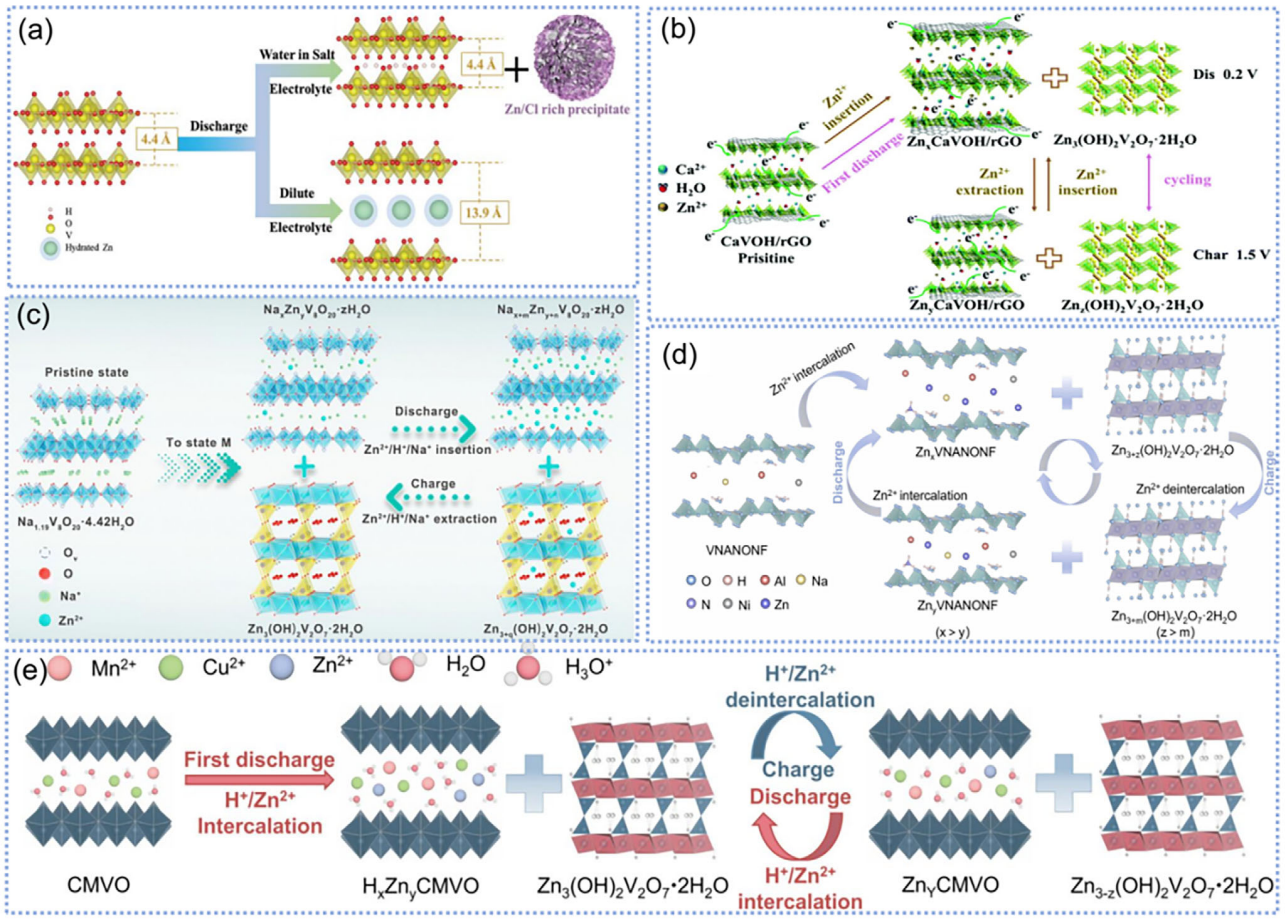
(a) Schematic illustration of the dynamic structural evolution of a V_2_O_5_ cathode in different electrolyte systems [219]. Copyright 2021, Wiley‐VCH GmbH. (b) Schematic illustration of the Zn^2+^ storage mechanism in the CaVOH/rGO composite electrode [220]. Copyright 2021, the Partner Organisations. (c) Schematic diagram illustrating the energy storage mechanism of Vo‐NVO‐2 [221]. Copyright 2023, the Royal Society of Chemistry. (d) Schematic illustration of the energy storage mechanism in the VNANONF electrode [222]. Copyright 2025, Elsevier. (e) Schematic illustration of the Zn^2+^/H^+^ co‐(de)intercalation mechanism during charge and discharge processes [223]. Copyright 2024, Wiley‐VCH GmbH.

Vanadium‐based oxides: Vanadium oxides, particularly V_2_O_5_, are regarded as promising cathode materials due to their high theoretical capacity [210, 211, 212, 213, 214, 215]. However, their practical application is significantly hampered by intrinsically low electronic conductivity and pronounced structural degradation during repeated charge‐discharge cycles [216, 217, 218, 219]. Notably, the intercalation of conductive 2D materials, such as graphene or MXenes into V_2_O_5_ layers, has been demonstrated as an effective strategy to markedly enhance electron transport, stabilize the host layered architecture, and expand the interlayer channels, thereby facilitating accelerated Zn^2^^+^ diffusion kinetics [220, 221, 222, 223]. This synergistic interaction not only significantly enhances the rate capability but also effectively suppresses the dissolution of vanadium species, thereby greatly improving the long‐term cycling stability of the electrodes. For example, Tang et al. conducted a systematic investigation into the detrimental effects of H_2_O/Zn^2+^ co‐insertion in the V_2_O_5_ cathode (Figure 8a), spanning from the microscopic scale to the full electrode level [219]. Their work demonstrated that water‐in‐salt electrolytes (WiSE) facilitate a shift toward an H^+^ insertion/extraction‐dominated charge storage mechanism in V_2_O_5_ [219]. This transition significantly alleviates mechanical stress within the cathode and enhances its structural stability. Hu et al. developed a composite material featuring a “double guarantee mechanism” through the integration of Ca^2+^ ‐intercalated hydrated vanadium oxide (V_2_O_5_·nH_2_O, abbreviated as VOH) with rGO (Figure 8b) [220]. The pre‐intercalated Ca^2+^ ions not only expanded the interlayer spacing but also significantly reduced electrostatic interactions, thereby enhancing the structural reversibility of the vanadium oxide host [220]. Simultaneously, the incorporated rGO improved electrical conductivity and provided mechanical stability during repeated charge/discharge cycles, contributing to outstanding electrochemical performance [220]. The XRD pattern shows that the strongest peak of pristine VOH is located at 7.73°, corresponding to an interlayer spacing of 11.4 Å. After incorporation of rGO (VOH/rGO), this peak shifts to 6.94°, indicating an expanded interlayer spacing of 12.7 Å.Upon further intercalation of Ca^2+^ (CaVOH/rGO), the strongest peak shifts further to 6.63°, corresponding to an even larger interlayer spacing of 13.3 Å. This directly demonstrates that Ca^2+^ ions successfully act as “pillars” to expand the layered structure of VOH. Consequently, the CaVOH/rGO//Zn battery delivers an exceptional specific capacity of 409 mA h g^−1^ at 0.05 A g^−1^. Moreover, it demonstrates remarkable long‐term cyclability, retaining over 90% of its capacity (299 mA h g^−1^) after 2000 cycles at 4.0 A g^−1^, along with an impressive energy density of 381 W h kg^−1^ at a power density of 48 W kg^−1^ [220]. Wu et al. utilized a facile hydrothermal approach to fabricate Na_1.19_V_8_O_20_·4.42H_2_O nanosheets enriched with oxygen vacancies and pre‐intercalated Na^+^, demonstrating their potential as a high‐performance cathode for AZIBs (Figure 8c) [222]. The material features expanded interlayer spacing, accelerated reaction kinetics, and exceptional structural durability. These improvements are attributed to the synergistic effects of oxygen vacancies, Na^+^ pre‐intercalation, and abundant structural water, which collectively enhance electrochemical activity, electronic conductivity, and Zn^2+^ diffusion dynamics [170]. Ding et al. proposed an innovative synergistic strategy involving the incorporation of multiple species (Na^+^, Al^3+^, Ni^2+^, NH_4_^+^, and F^−^) into the interlayers of V_2_O_5_ to achieve a high‐entropy configuration (Figure 8d) [222]. The cationic doping not only serves as structural pillars to enhance the stability of the V_2_O_5_ framework but also markedly reduces desolvation energy loss and accelerates intercalation kinetics [222]. Furthermore, the introduction of F^−^ anions strengthens the binding interactions between anions and cations, thereby improving structural integrity and promoting both electronic and ionic conductivity [222]. Wang et al. designed a bimetallic Cu/Mn‐codoped layered V_2_O_5_ cathode via a hydrothermal method (Figure 8e) [223]. The pre‐intercalated Cu^2+^ ions function as structural pillars that stabilize the vanadium oxide (V–O) layers, forming well‐defined 2D channels for rapid Zn^2+^ diffusion. Meanwhile, the incorporation of Mn ions further expands the interlayer spacing and increases the concentration of oxygen defects, thereby significantly enhancing Zn^2+^ diffusion kinetics [228]. XRD shows that the (001) interlayer spacing of Mn^2+^‐doped CMVO is 13.48 Å, whereas that of Cu^2+^‐only CVO is 11.51 Å. This is because Mn^2+^ has a larger ionic radius than Cu^2+^, and its intercalation further expands the V‐O interlayer spacing, providing a more spacious and rapid diffusion pathway for the large hydrated Zn^2+^ ions ([Zn(H_2_O)_6_]^2+^).

**FIGURE 9:**
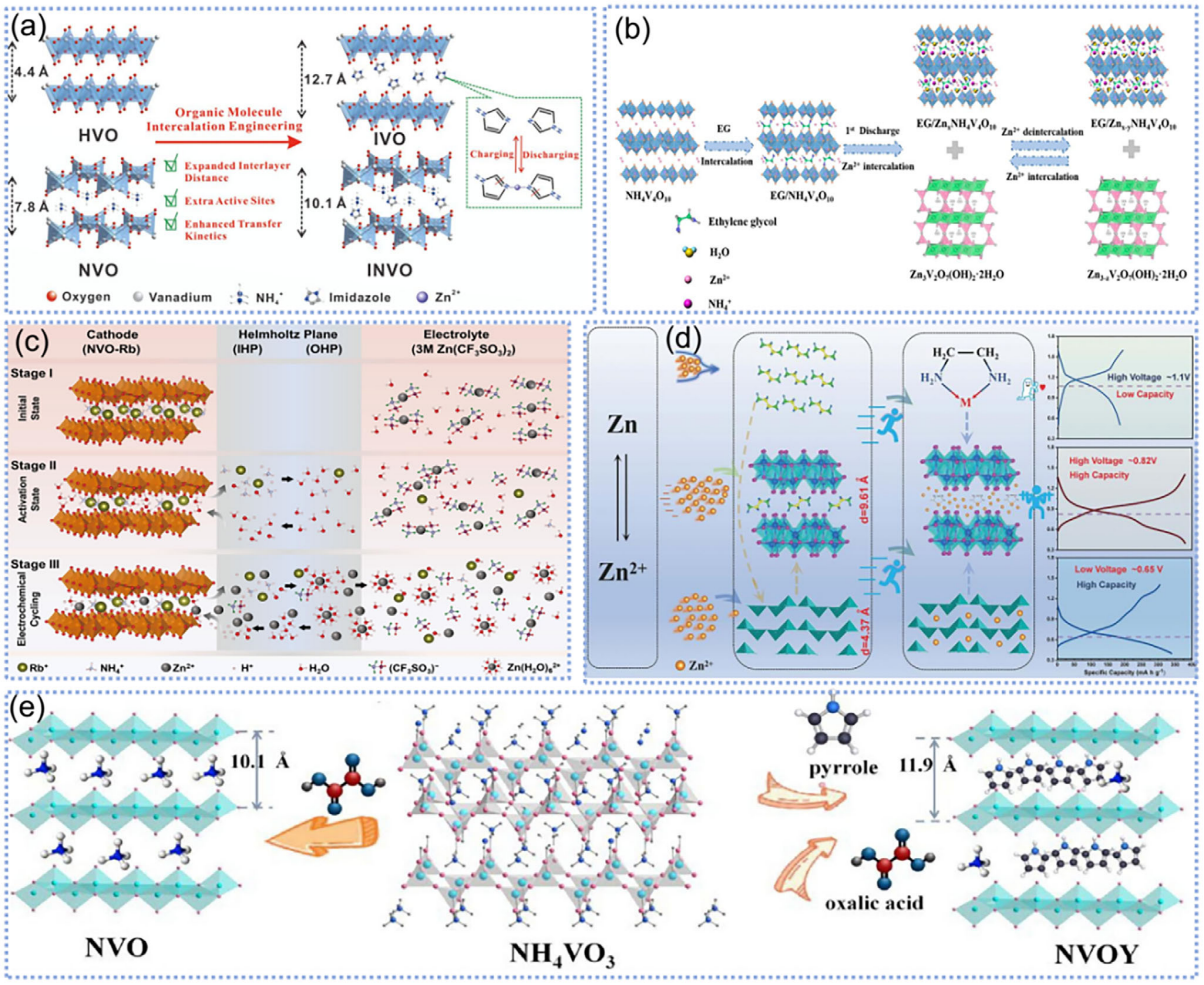
(a) Schematic illustration of the proposed imidazole intercalation strategy and its associated advantages [226]. Copyright 2024, American Chemical Society. (b) Schematic diagram of the Zn^2+^ storage mechanism in EG‐NVO [227]. Copyright 2024, American Chemical Society. (c) Schematic illustration of Rb^+^ deintercalation and its effect on the solvation structure of Zn(H_2_O)_6_^2+^ in the Zn//Zn(CF_3_SO_3_)_2_//NVO‐Rb battery system at various operational stages [228]. Copyright 2024, American Chemical Society. (d) Schematic illustration of the organic–inorganic hybrid cathode composed of ethylenediamine (EDA) and vanadium oxide, delivering high capacity and high operating voltage [229]. Copyright 2022, Wiley‐VCH GmbH. (e) Schematic diagram illustrating the fabrication process of the polypyrrole intercalated ammonium vanadate (NVOY) electrode [230]. Copyright 2024, Elsevier.

Compared with conventional metal ions, ammonium ions (NH_4_^+^) serve as a lightweight alternative that enhances specific capacity. Their pillaring effect not only enlarges the interlayer spacing to facilitate rapid Zn^2+^ diffusion but also stabilizes the host structure through hydrogen bonding with V–O layers [224, 225, 226]. This dual functionality effectively mitigates volume variation during cycling and preserves high ionic conductivity. For example, Tang et al. proposed an organic imidazole intercalation strategy and employed V_2_O_5_ and NH_4_V_3_O_8_ (NVO) as model materials to validate the effectiveness of imidazole in enhancing the zinc storage performance of vanadium‐based cathodes (Figure 9a) [226]. The intercalated imidazole molecules not only act as structural pillars to expand the interlayer spacing and enhance structural stability but also provide additional coordination sites for zinc ion storage through the coordination interaction between Zn^2+^ and C–N groups [227]. XRD results indicate that successful intercalation of imidazole expands the interlayer spacing, confirming that imidazole molecules act as “pillars” to enlarge the interlayer distance and stabilize the crystal structure. DFT calculations further show that after imidazole intercalation, the adsorption energy of Zn^2+^ in the material is significantly enhanced, demonstrating that imidazole molecules greatly improve the material's binding affinity for Zn^2+^. Chen et al. developed a cathode material for AZIBs based on NH_4_V_4_O_10_ nanoflowers intercalated with ethylene glycol (Figure 9b) [227]. The insertion of ethylene glycol expanded the interlayer spacing by approximately 23%, creating wide diffusion channels for Zn^2+^ transport [227]. Simultaneously, the presence of EG induced the self‐assembly of ultrathin nanosheets into nanoflower‐like architectures, which substantially increased the exposure of active sites and facilitated ion and electron transfer [227]. Wang et al. introduced rubidium cations (Rb^+^, 1.47 Å) to synthesize Rb^+^‐preintercalated NH_4_V_4_O_10_ (Figure 9c), significantly enhancing the long‐term cycling stability of NVO‐based cathodes for AZIBs [227]. Moreover, particular attention has been directed toward the role of deintercalated Rb^+^ ions in modulating the electrochemical behavior of the aqueous electrolyte and promoting uniform zinc deposition on the anode [228]. Ma et al. reported an organic–inorganic hybrid cathode material of C_2_H_8_N_2_V_7_O_16_ for ZIBs (Figure 9d) [229]. By leveraging the high capacity of vanadium oxide and the elevated working voltage contributed by ethylenediamine (EDA), this hybrid cathode delivers a high average operating voltage of 0.82 V, along with superior specific capacity and extended cycling stability [229]. The EDA molecules not only expand the interlayer spacing of the vanadium oxide framework, thereby facilitating Zn^2^^+^ mobility within the V‐O layers, but also function as bidentate chelating ligands that actively participate in zinc ion storage [229]. Gong et al. proposed a novel strategy involving the introduction of polypyrrole (PPy) to partially replace excess ammonium ions in the interlayer of NH_4_V_4_O_10_, thereby enhancing the electrochemical performance of AZIBs [230]. The synergistic effect of NH_4_^+^ removal and PPy incorporation results in expanded interlayer spacing and the generation of abundant oxygen vacancies, which collectively provide additional active sites for zinc ion migration, enhance interfacial electronic/ionic conductivity, and stabilize the host structure (Figure 9e) [230]. Furthermore, the PPy polymer, along with the remaining NH_4_^+^ ions, functions as a structural pillar to support the layered framework and mitigates electrostatic repulsion between Zn^2+^ ions and the host material, thereby accelerating reaction kinetics [230]. FTIR spectroscopy reveals a redshift in the characteristic V–O bond peak, which is attributed to the presence of oxygen vacancies. Furthermore, GITT measurements confirm that the introduction of additional oxygen vacancies enhances electrical conductivity and accelerates reaction kinetics. As anticipated, the resulting polypyrrole‐intercalated ammonium vanadate cathode exhibits superior performance with a high capacity of 431.9 mAh g^−1^ at 0.5 A g^−1^ and excellent long‐term cyclability (219.1 mAh g^−1^ at 20 A g^−1^ with 78% retention over 1500 cycles) [230]. These strategies work synergistically to boost intrinsic and interfacial conductivity, stabilize the host lattice, accelerate Zn^2+^ transport, and inhibit vanadium dissolution in electrolytes, substantially elevating the material's rate capability and cycling durability over long cycles.

**FIGURE 10:**
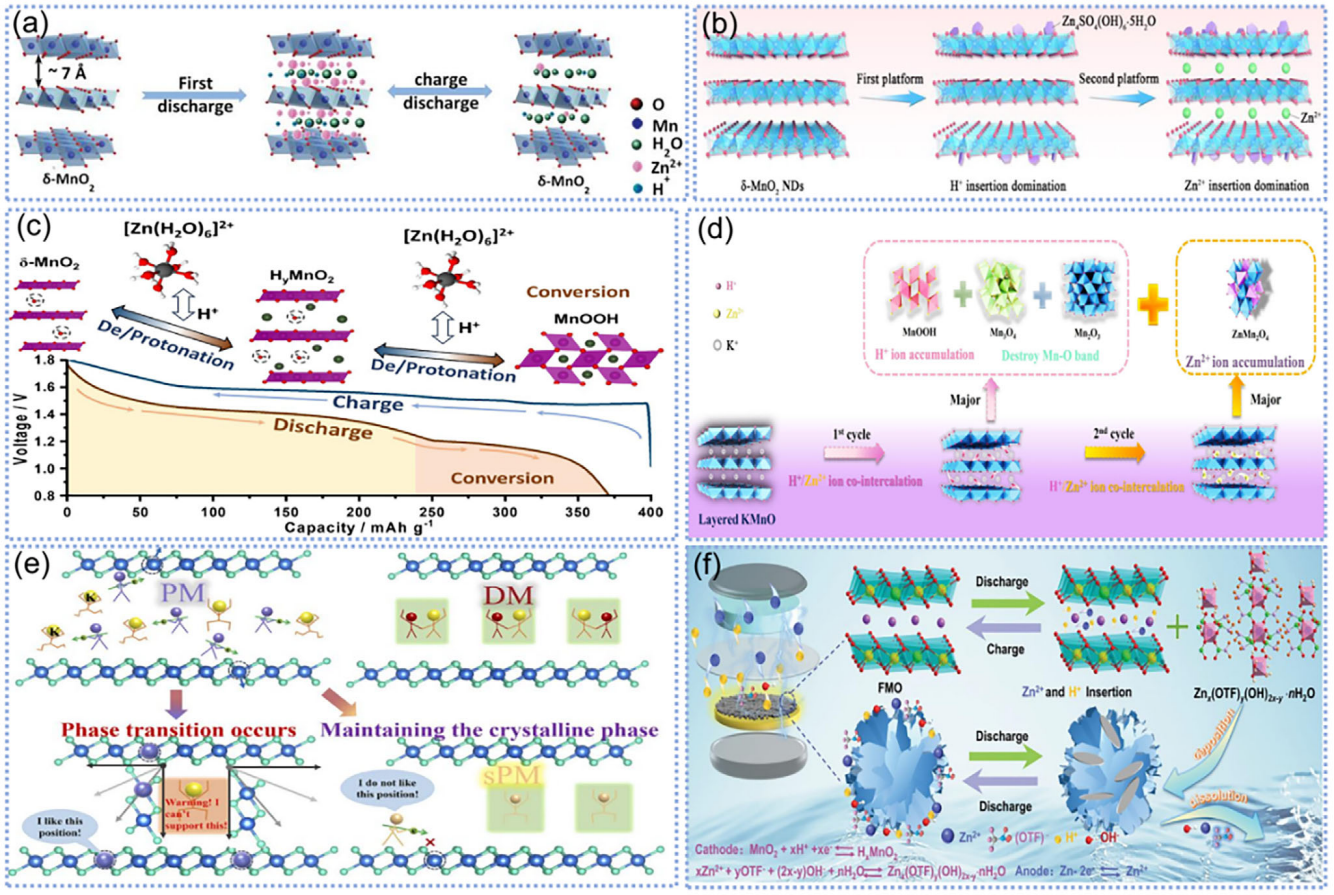
(a) Schematic illustration of δ‐MnO_2_ and the zinc storage mechanisms [236]. Copyright 2020, Wiley‐VCH GmbH. (b) Schematic illustration of the electrochemical energy storage mechanism of the δ‐MnO_2_ NDs electrode in ZIBs [237]. Copyright 2022, Elsevier. (c) Schematic representation of the working mechanism of the Zn‐MnO_2_/rGO cell after activation, alongside its corresponding voltage profile at the 10th cycle (0.1C) showing the discharge and reversed charge processes [238]. Copyright 2024, Elsevier. (d) Schematic of the Zn^2+^/H^+^ co‐intercalation mechanism in a layered K^+^‐pillared layered MnO_2_ electrode [239]. Copyright 2024, Elsevier. (e) Schematic illustrating the phase transformation process of δ‐MnO_2_ during transition metal ion intercalation [240]. Copyright 2025, Wiley‐VCH GmbH. (f) Schematic illustration of the reaction mechanism in an aqueous Zn/Co_x_MnO_2_ battery [198]. Copyright 2025, Wiley‐VCH GmbH.

Manganese‐based oxides: 2D manganese‐based oxides, particularly δ‐MnO_2_, have attracted considerable interest as cathode materials for AZIBs owing to their high operating voltage and environmental friendliness [231, 232, 233]. However, their practical deployment is largely hindered by irreversible Mn^3+^ dissolution and Jahn‐Teller distortion, which collectively contribute to rapid capacity fading and structural degradation [201, 224, 225, 226, 227, 228, 229, 230, 231, 232, 233, 234, 235]. By compositing with conductive 2D substrates (e.g., rGO or MXene), the dissolution of Mn ions is suppressed through strong interfacial interaction, while the conductive network enhances charge transfer and structural integrity [236, 237, 238, 239, 240]. Moreover, 2D additives help stabilize the MnO_2_ crystal structure, reducing phase transformation during Zn^2+^ insertion/extraction. For example, Jiang presented a binder‐free δ‐MnO_2_ cathode that enables a favorable “layered‐to‐layered” Zn^2+^ storage mechanism (Figure 10a) [236]. The layered δ‐MnO_2_ possesses a wide interlayer spacing that enables reversible Zn^2+^ insertion, fast ion diffusion, and rapid electron transport [236]. Tang et al. developed layered δ‐MnO_2_ nanodisks (NDs) through a simple redox reaction for use as a high‐performance cathode material in AZIBs [237]. During the first discharge plateau, H^+^ insertion into the δ‐MnO_2_ NDs electrode occurs, leading to a gradual increase in local OH^−^ concentration near the electrode surface and subsequent formation of Zn_4_SO_4_(OH)_6_·xH_2_O (Figure 10b) [237]. As H^+^ concentration decreases, subsequent Zn^2+^ insertion takes place, giving rise to the second discharge plateau [237]. The nanosized structure offers abundant active sites, enhanced ion/electron diffusion kinetics, and reduced mechanical stress during charge/discharge cycles, leading to improved specific capacity, rate capability, and cycling stability [237]. Zhanadilov et al. proposed a layered birnessite‐type δ‐K_0.32_MnO_2_·0.15H_2_O as a cathode material for AZIBs (Figure 10c) [238]. This structure is well‐suited for Zn^2+^ intercalation within the Mn‐O interlayer, which exhibited excellent rate performance up to 10 C, facilitated by reduced graphene oxide (rGO) [238]. Mechanistic studies indicate that the charge storage proceeds via a conversion reaction involving protonation/deprotonation of MnO_2_, ultimately leading to amorphization after prolonged cycling (Figure 10c) [238]. Song et al. developed a high‐capacity K^+^‐pillared birnessite‐type δ‐MnO_2_ through an interfacial redox reaction between KMnO_4_ and sulfur at the aqueous/toluene interface (Figure 10d) [239]. The ultrasmall nanosphere architecture shortens ion transport pathways, while content‐tuned pre‐intercalation optimizes lattice spacing, facilitating rapid Zn^2^^+^ diffusion and storage [196]. More importantly, the size effect promotes the formation of a more amorphous Zn_4_SO_4_(OH)_6_·5H_2_O (ZSH) phase with higher water solubility compared to larger‐sized structures [239]. These features collectively enhance intrinsic conductivity and structural stability, effectively mitigate ZSH‐related side effects, and significantly improve high‐rate long‐term cycling performance. Notably, doping MnO_2_ with metal ions introduces impurity energy levels into the electronic structure, which can enhance electrical conductivity and improve potassium storage performance. Wang et al. introduced various metal ions (e.g., Mo^6+^, V^5+^, W^6+^, Cu^2+^) into layered MnO_2_, which induced lattice distortion without phase transition (Figure 10e) [240]. Mo‐doped δ‐MnO_2_ with crystal defects generated abundant oxygen vacancies, increasing active sites and delivering an ultrahigh specific capacity of 110 mAh g^−1^ at 0.1 A g^−1^ [240]. Moreover, these metal ions could generate strong point electric fields within the interlayers of δ‐MnO_2_, thus preventing K^+^ escape. Their work focused on the correlation between the magnetic moments of transition metal ions and structural transformations in MnO_2_, offering targeted theoretical insights for the performance optimization and synthesis of MnO_2_‐based materials. Li et al. synthesized δ‐MnO_2_ nanosheets via a surfactant‐assisted precipitation method and systematically investigated the effects of Fe^3+^ and Co^2+^ doping on the electrochemical properties and reaction mechanisms [198]. The substitution of Mn by these metal cations forms M‐O bonds, which enhance the structural stability and redox activity of MnO_2_. Specifically, Fe doping modulates the interaction between Zn^2^^+^/H^+^ and the host structure, suppressing the formation of by‐products such as ZnMn_2_O_4_ (ZMO), while Co doping facilitates rapid Zn^2^^+^ diffusion (Figure 10f) [198]. Their study deepens the fundamental understanding of Zn‐ion storage mechanisms and provides an effective strategy for enhancing the performance of MnO_2_‐based cathodes.

**FIGURE 11:**
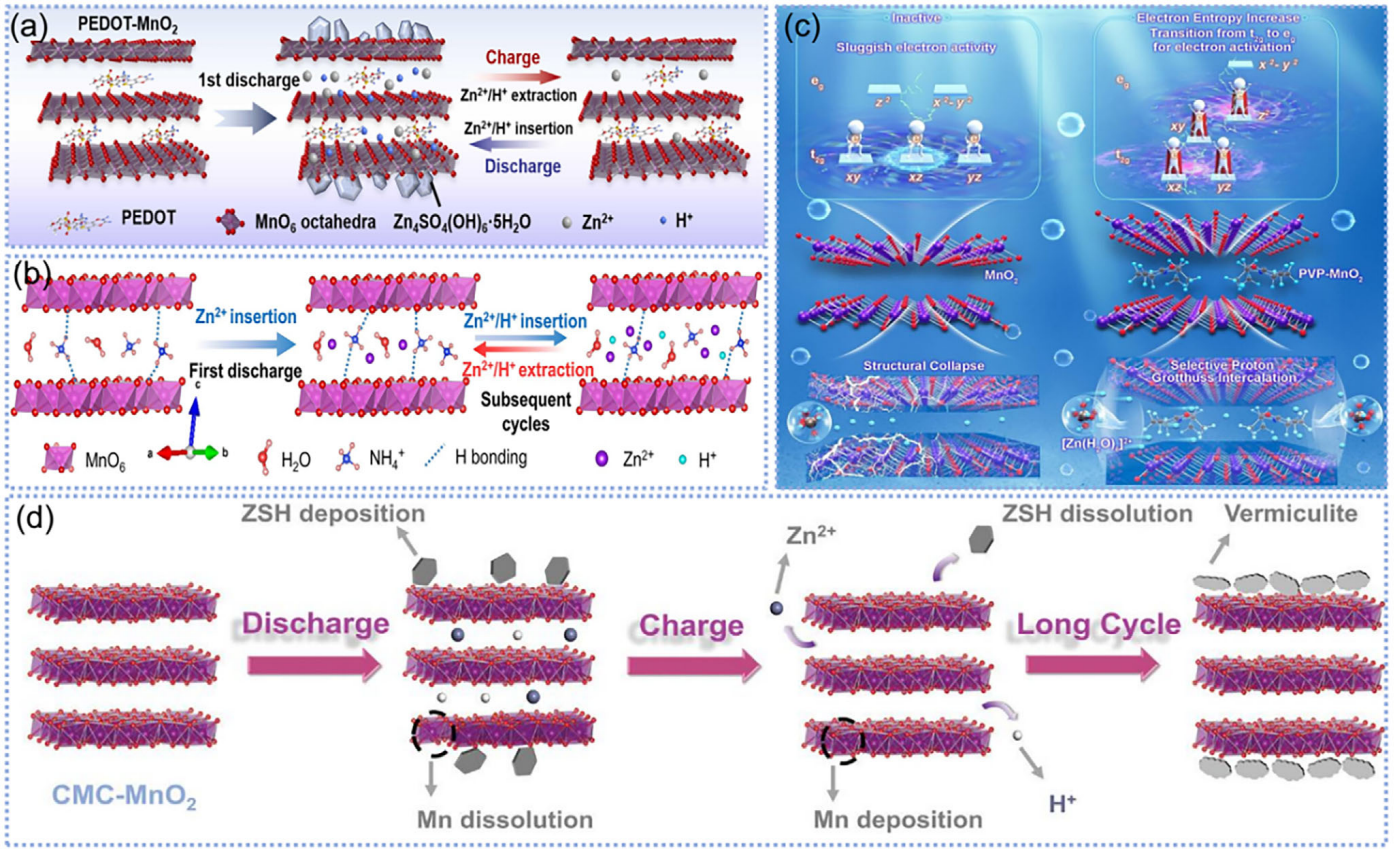
(a) Schematic illustration of the energy storage mechanism of PEDOT‐MnO_2_‐2 [243]. Copyright 2023, Elsevier. (b) Schematic of the Zn^2+^/H^+^ co‐(de)intercalation mechanism in (NH_4_)_x_MnO_2_ cathode [244]. Copyright 2022, Wiley‐VCH GmbH. (c) The optimized energy storage mechanism involves Mn electron entropy enhancement and selective proton Grotthuss intercalation of interlayer configuration optimization [245]. Copyright 2023, Wiley‐VCH GmbH. (d) Schematic of the Zn^2+^ ion intercalation mechanism in the CMC‐MnO_2_ cathode [246]. Copyright 2025, Wiley‐VCH GmbH.

The pre‐intercalated organic molecules function as structural pillars between the material layers. This configuration not only significantly expands the interlayer spacing, but also enhances the stability of the crystal structure during repeated Zn^2+^ insertion and extraction cycles [241, 242]. Furthermore, the introduction of a conductive polymer into the host crystal through pre‐intercalation simultaneously addresses two critical challenges: it substantially reduces both the intrinsic bulk resistance of the material and the interfacial resistance, thereby improving overall electrochemical performance and structural durability [241, 242, 243, 244, 245, 246]. For example, Chen et al. creatively synthesized a PEDOT‐intercalated MnO_2_ composite (PEDOT‐MnO_2_) through a redox precipitation reaction involving KMnO_4_, MnSO_4_, and EDOT [243]. The pre‐intercalated PEDOT not only enlarges the interlayer spacing but also significantly enhances the electrical conductivity of MnO_2_, thereby facilitating rapid electron transfer and efficient Zn^2+^ diffusion (Figure 11a) [243]. Moreover, PEDOT acts as “structural pillars” that stabilize the MnO_2_ crystal framework during repeated charge/discharge processes, improving structural durability and cycling performance [245]. Wang et al. reported a preintercalated (NH_4_)_x_MnO_2_ cathode featuring ammonium ions (NH_4_^+^) between the MnO_2_ interlayers [244]. The introduction of NH_4_^+^, which has a low molar mass, effectively enlarges the interlayer spacing, thereby facilitating faster ion diffusion (Figure 11b) [244]. Simultaneously, these ions act as “structural pillars” to mitigate layer collapse during cycling. Furthermore, the hydrogen bonding interaction between NH_4_^+^ and the host MnO_2_ framework enhances charge transfer kinetics and contributes to the overall structural stability of the cathode material [244]. Therefore, the (NH_4_)_x_MnO_2_ cathode exhibits a high specific capacity of 228.7 mAh g^−1^ at 0.3 A g^−1^, maintains superior high‐rate performance with 122.5 mAh g^−1^ at 3.0 A g^−1^, and demonstrates outstanding cycling stability, retaining its capacity over 1500 cycles at 2.0 A g^−1^ without significant decay [244]. Zhang et al. proposed a terrace‐shaped δ‐MnO_2_ hybrid superlattice through the pre‐intercalation of polyvinylpyrrolidone (PVP) [245]. This hybrid superlattice promotes selective Grotthuss‐type proton (H^+^) intercalation, while hindering the desolvation of hydrated [Zn(H_2_O)_6_]^2+^ ions via interlayer spatial confinement and charge redistribution, resulting in an elevated transport energy barrier (Figure 11c) [245]. The enhanced Mn electron entropy and selectively accelerated proton transport kinetics collectively lead to a significant improvement in specific capacity and rate performance. Meanwhile, the suppressed diffusion of [Zn(H_2_O)_6_]^2+^ ions contributes to superior cycling stability by mitigating structural degradation (Figure 11c) [245]. Accordingly, the PVP‐MnO_2_ hybrid superlattice demonstrates outstanding electrochemical performance, delivering a high specific capacity of 317.2 mAh g^−1^ at 0.125 A g^−1^, a remarkable rate capability of 106.1 mAh g^−1^ at 12.5 A g^−1^, and exceptional long‐term cycling stability with nearly 100% capacity retention after 20 000 cycles at 10 A g^−1^ [245]. Zhang et al. synthesized small‐sized amorphous MnO_2_ nanosheets via a facile hydrothermal method using carboxymethylcellulose sodium (CMC) as a capping agent [246]. The abundant carboxymethylcellulose groups in CMC selectively bind to the edges of [MnO_6_] units, effectively restricting crystal growth and resulting in MnO_2_ with reduced dimensions (Figure 11d) [246]. The combination of ultrasmall nanosheet morphology and amorphous nature endows the material with abundant structural defects and shortened ion diffusion pathways, thereby significantly enhancing electrochemical performance. Furthermore, the introduced oxygen anions help stabilize Mn^3+^ and suppress its disproportionation reaction, particularly beneficial for high‐spin Mn^3+^ [246]. These synergistic modifications inhibit side reactions, optimize ion conduction paths, improve electronic conductivity, and maintain structural stability of the host framework, leading to remarkable electrochemical performance.

**FIGURE 12:**
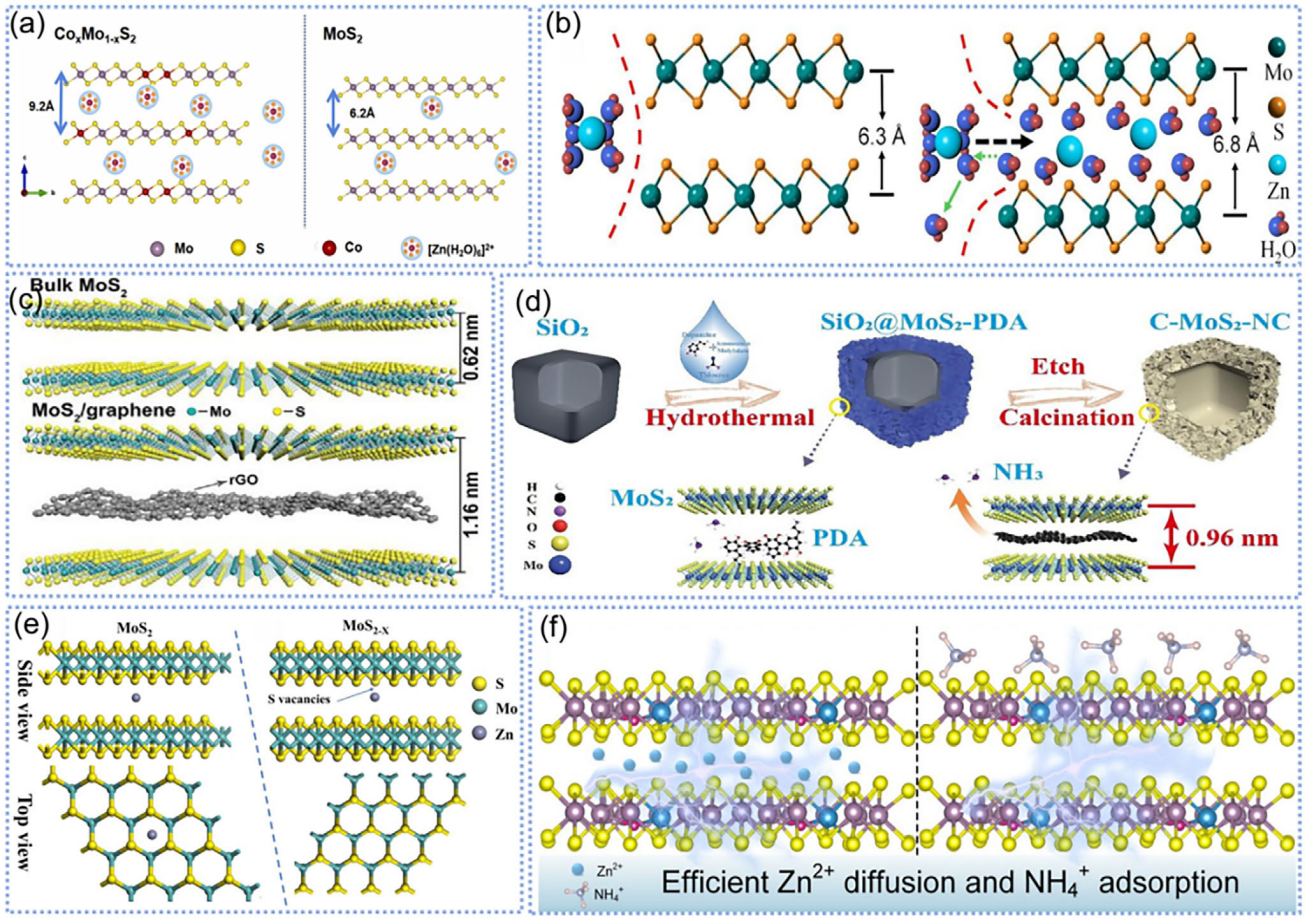
(a) Schematic illustration of Zn(H_2_O)_6_^2+^ insertion into and extraction from MoS_2_ and Co_x_Mo_1‐x_S_2_ [248]. Copyright 2022, Elsevier. (b) Schematic of hydrated Zn^2+^ insertion into dehydrated (left) and hydrated (right) MoS_2_ [249]. Copyright 2021, Elsevier. (c) Schematic of crystal structures of bulk MoS_2_ and MoS_2_/graphene [250]. Copyright 2021, Wiley‐VCH GmbH. (d) Schematic of the C‐MoS_2_‐NC synthesis process [251]. Copyright 2022, Elsevier. (e) Side and top views of Zn^2+^ storage in MoS_2_ and MoS_2‐x_ layers, respectively [252]. Copyright 2023, Elsevier. (f) Schematic diagram of optimizing the storage of Zn^2+^ and NH^4+^ with Mo_0.99_W0_.01_O_0.17_S_1.83‐x_ [253]. Copyright 2025, American Chemical Society.

Transition metal sulfur chalcogenides: 2D transition metal chalcogenides (TMCs), such as MoS_2_, VS_2_, VSe_2_ and TiS_2_, have attracted growing interest as cathode materials for AZIBs due to their unique layered structures, high theoretical capacity, and tunable electronic properties [127, 247, 248, 249, 250, 251, 252, 253, 254]. These materials typically exhibit large interlayer spacing and abundant active sites, which are conducive to the reversible intercalation and deintercalation of Zn^2+^ ions. However, the practical deployment of these materials is hindered by several critical challenges of intrinsically low electrical conductivity, structural degradation upon repeated cycling, and sluggish Zn^2+^ diffusion kinetics, which collectively compromise rate capability, cycling stability, and overall electrochemical performance. To address these issues, various modification strategies have been developed, including interlayer engineering, anion/cation doping, and hybridization with conductive materials (e.g., rGO, carbon nanotubes, MXenes, polymers) [244, 248]. These functional optimizations not only improve the electrochemical performance of TMS‐based cathodes but also provide deeper insights into the material design strategies for high‐performance energy storage systems. For example, Zhou et al. report a strategy of incorporating Co into MoS_2_ to form Co_x_Mo_1‐x_S_2_ nanosheets, which feature abundant dislocation defects and expanded interlayer spacing (Figure 12a) [248]. While pristine MoS_2_ suffers from poor Zn^2+^ diffusivity and low specific capacity due to its narrow interlayer spacing, the Co doping strategy significantly enhances the diffusion kinetics of Zn^2+^ ions [248]. The use of Co_x_Mo_1‐x_S_2_ nanosheets as the cathode enhances the ZIB capacity fivefold by effectively lowering the Zn^2+^ embedding energy barrier and consequently boosting its internal diffusion rate [248]. Liu et al. revealed the critical role of crystal water in enhancing the Zn^2+^ intercalation kinetics of layered MoS_2_ (Figure 12b) [249]. The incorporated water molecules act as structural pillars, expanding the interlayer spacing and improving the host material's hydrophilicity. This modification significantly boosts the charge‐transfer kinetics and Zn^2+^ diffusivity. As a result, the hydrated MoS_2_ cathode achieved efficient Zn^2+^ storage, delivering a high specific capacity of 182 mAh g^−1^ at 0.1 A g^−1^ along with superior rate capability and cycling stability. Li et al. developed a high‐performance cathode for AZIBs by constructing a sandwich‐like MoS_2_/graphene nanocomposite [250]. The pioneering intercalation of rGO expanded the MoS_2_ interlayer spacing to 1.16 nm (Figure 12c), and the combined effects of the 1T phase and rGO's oxygen groups enhanced hydrophilicity and suppressed layer stacking [250]. This design yielded exceptional rate capability (285.4 mAh g^−1^ at 0.05 A g^−1^ vs. 141.6 mAh g^−1^ at 5 A g^−1^) and cycling stability (88.2% retention after 1800 cycles) [250]. By employing a template‐assisted wet‐chemistry approach and pyrolysis (Figure 12d), Li et al. fabricated MoS_2_‐nanosheet‐assembled nanocages (C‐MoS_2_‐NC) from the bottom up [251]. The N‐doped carbon (NC) motifs inserted during synthesis induce a drastic interlayer expansion from 0.62 to 0.96 nm [260]. The NC motifs offer abundant channels for mass and electron transport, while the cage‐shaped design concurrently inhibits nanosheet stacking and buffers volume changes during (dis)charge, enabling its high performance as an AZIB cathode [251]. By incorporating sulfur vacancies and N‐doped carbon nanotubes (NCNTs) into MoS_2_ to form MoS_2‐x_@NCNTs (Figure 12e), Zhao et al. significantly lowered the Zn^2+^ diffusion energy barrier and enhanced the kinetics, thereby achieving superior rate capability and long‐term cycling stability [252]. Long et al. engineered Mo_0.99_W_0.01_O_0.17_S_1.83‐x_ via W/O co‐doping (Figure 12f), revealing a synergistic mechanism where lattice distortion stabilizes the 1T phase, enhances conductivity via charge redistribution, and exposes active sites, collectively boosting carrier migration [253]. Overall, the strategic integration of 2D materials with conventional cathode hosts represents a promising pathway toward developing high‐performance AZIBs with improved conductivity, structural stability, and electrochemical reactivity.

**FIGURE 13:**
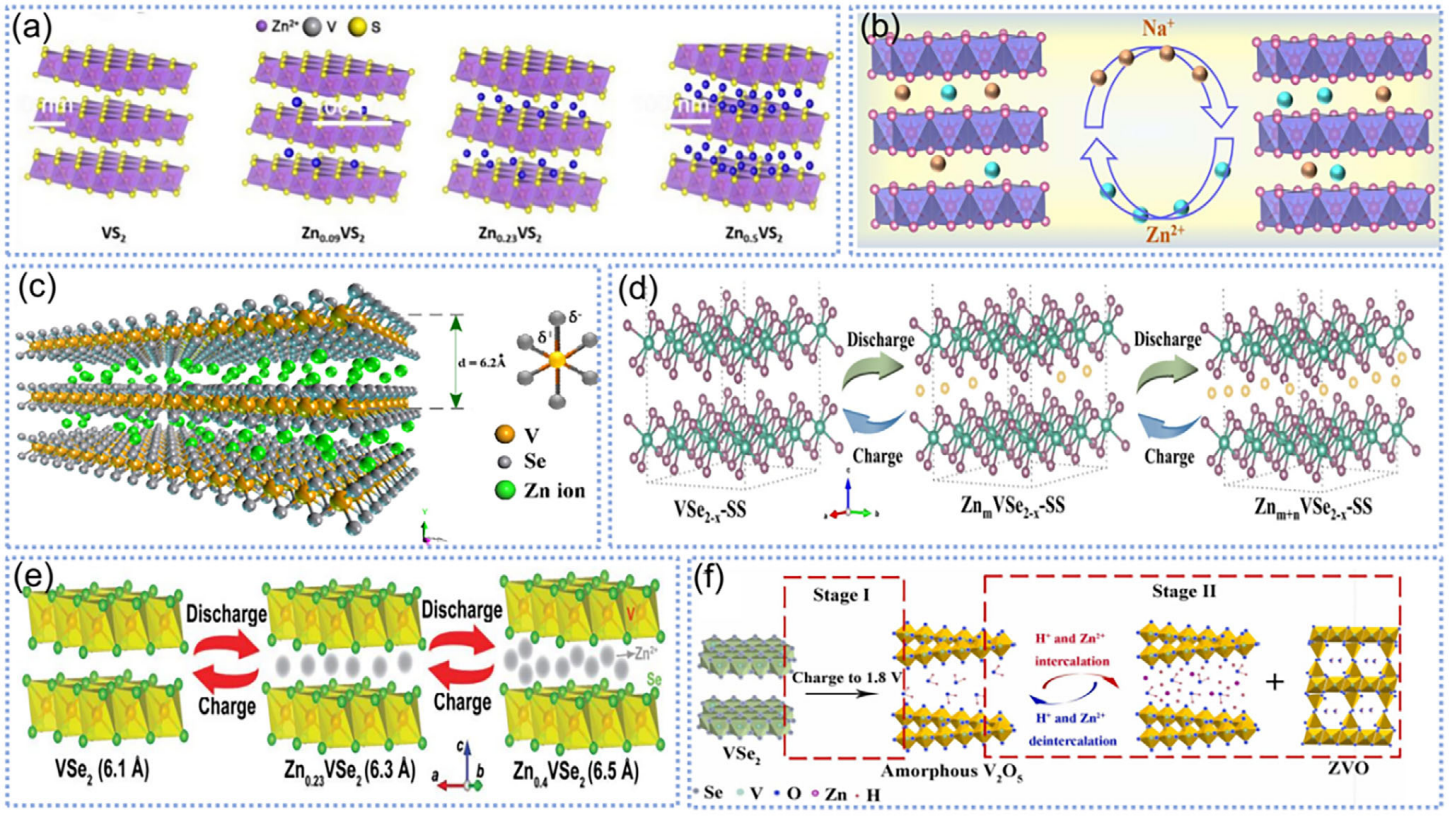
(a) Schematic of the gradual Zn^2+^ intercalation into VS_2_ [254]. Copyright 2024, American Chemical Society. (b) Schematic illustration Zn^2+^ and Na^+^ storage mechanism inVS_2_ [255]. Copyright 2025, American Chemical Society. (c) Schematic illustration of Zn^2+^ storage in rGO‐VSe_2_ nanohybrid [256]. Copyright 2021, Elsevier. (d) Schematic illustration of the two‐step Zn^2+^ insertion/extraction process in VSe_2‐x_‐SS [257]. Copyright 2021, American Chemical Society. (e) Schematic illustration of the two‐step Zn^2+^ (de)intercalation process in VSe_2_ cathode [258]. Copyright 2020, Wiley‐VCH GmbH. (f) Schematic illustration of the electrochemical oxidation of VSe_2_ to amorphous V_2_O_5_ and the subsequent reversible Zn^2+^ intercalation [259]. Copyright 2024, Elsevier.

As a typical member of TMCs, VS_2_ crystallizes in a hexagonal system with a lamellar structure analogous to graphite [254, 255, 256, 257, 258, 259]. Its crystal structure features a sandwich‐like configuration, where a vanadium layer is sandwiched between two sulfur layers. Each vanadium atom is octahedrally coordinated by six sulfur atoms, bonded via covalent interactions. A notable feature of VS_2_ is its large interlayer spacing (5.76 Å), which facilitates the facile insertion and extraction of zinc ions and even their solvated species from the electrolyte [257, 258, 259]. The storage mechanism in VS_2_ involves highly reversible phase evolution. As illustrated in Figure 13a, the storage process involves multi‐step Zn^2+^ intercalation, leading to the sequential formation of Zn_x_VS_2_ [254]. Liu et al. synthesized VS_2_ nanosheets directly on a carbon cloth (VS_2_/CC) substrate via a one‐step hydrothermal method [255]. This in situ growth strategy ensures strong adhesion between the VS_2_ nanosheets and the conductive CC, which is essential for improving charge transport kinetics and maintaining structural integrity of the electrode. The combination of the conductive CC substrate and the Zn^2+^/Na^+^ co‐intercalation strategy synergistically promotes rapid ion diffusion and enhances the structural stability of VS_2_ during cycling (Figure 13b) [255]. Similar to VS_2_, VSe_2_ exhibits a layered structure, featuring a V‐Se‐Se sandwich held by van der Waals forces (Figure 13c) [256]. With an interlayer spacing of 6.11 Å, it offers sufficient room for Zn^2+^ migration and storage [256]. The material's metallic character, arising from strong V^2+^‐V^2+^ electron coupling, grants it high conductivity. These properties collectively establish VSe_2_ as an attractive electrode candidate for ZIBs. To enhance the conductivity and activity of VSe_2_, Bai et al. proposed a strategy for the hydrothermal reduction synthesis of stainless‐steel‐supported VSe_2_ nanosheets with Se defects (VSe_2‐x_‐SS) [257]. The introduced defects not only adjust the Zn^2+^ adsorption energy but also render the subsequent adsorption/desorption process more reversible than that on the pristine VSe_2_‐SS. The Zn^2+^ storage mechanism involves surface adsorption/desorption and a two‐step intercalation process (Figure 13d) [257]. This self‐supporting electrode material effectively maintains structural integrity during cycling, which is responsible for the excellent cyclic performance. Wu et al. demonstrated a reversible but incomplete Zn^2+^ storage mechanism in VSe_2_ (Figure 13e), where partial zinc ions remain trapped after charging, and further identified a two‐step intercalation process during the battery's operation [258]. The VSe_2_ nanosheets demonstrate a discharge plateau between 1.0 and 0.7 V, delivering a specific capacity of 131.8 mAh g^−1^ at 0.1 A g^−1^ and a high energy density of 107.3 Wh kg^−1^ at a power density of 81.2 W kg^−1^. Notably, they also achieve outstanding cycling stability without any activation process, retaining 80.8% of their capacity after 500 cycles [258]. Wang et al. proposed an in situ electrochemical oxidation strategy to elevate the valence state of V atoms in VSe_2_, thereby enhancing its Zn^2+^ storage performance. This strategy induces an irreversible phase transformation during the first charge (1.35–1.8 V vs. Zn/Zn^2+^), converting the initial VSe_2_ into amorphous V_2_O_5_ (Figure 13f) [259]. The transformed product, featuring a high valence state and numerous active sites, subsequently acts as the durable host material for Zn^2+^ storage [259]. Overall, these approaches collectively improve ionic conductivity, structural stability, and cycling reversibility, providing valuable insights for designing high‐performance cathode materials.

**FIGURE 14:**
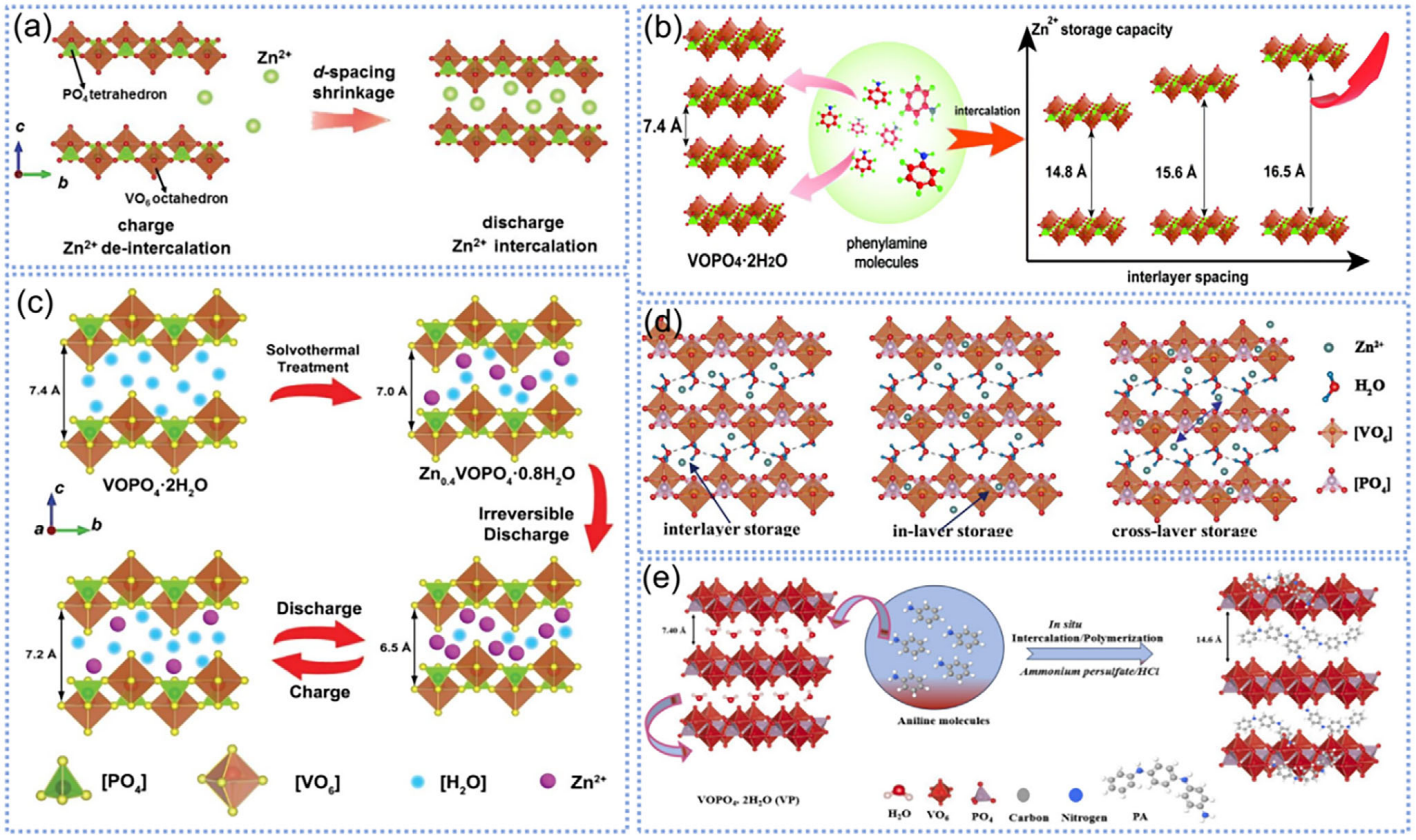
(a) Schematic diagram of the intercalation/deintercalation process in bilayer VOP nanosheets [260]. Copyright 2021, Wiley‐VCH GmbH. (b) Schematic illustrating of capacity modulation for zinc‐ion storage by PA‐intercalation engineering [261]. Copyright 2021, the Royal Society of Chemistry. (c) Schematic of topochemical transformation and d‐spacing evolution in layered phosphate across solvothermal synthesis and charge/discharge processes [262]. Copyright 2020, American Chemical Society. (d) Schematic diagram of Zn^2+^ storage and migration mechanisms in VO_d_‐VOPO_4_ [263]. Copyright 2024, Wiley‐VCH GmbH. (e) Synthesis mechanism of polyaniline‐intercalated VOPO_4_ by in situ intercalation/polymerization [264]. Copyright 2024, Elsevier.

2D layered VOPO_4_ possesses a structure defined by sheets stacked along the c‐axis direction with an interlayer spacing of 4.11 Å [260, 261, 262, 263, 264]. Its structure consists of alternating PO_4_ tetrahedra and distorted VO_5_ polyhedra within the ab‐plane (Figure 14a), connected via corner‐sharing oxygen atoms [260]. Compared with traditional Zn^2+^ intercalation, its application in ZIBs with a water‐in‐salt electrolyte reveals a hybrid mechanism combining reversible oxygen redox with Zn^2+^ insertion/extraction. Layered VOPO_4_ has emerged as a promising cathode material for rechargeable AZIBs owing to its unique layered framework and high discharge plateau [260]. However, its practical application is hindered by sluggish Zn^2+^ diffusion kinetics, limited specific capacity, and poor electrochemical stability. To address these challenges, a series of phenylamine (PA)‐intercalated VOPO_4_·2H_2_O materials with tunable interlayer spacings (14.8, 15.6, and 16.5 Å) were synthesized via a solvothermal method (Figure 14b) [261]. The optimal interlayer spacing of 16.5 Å facilitates rapid zinc‐ion diffusion, achieving an ultrahigh diffusion coefficient of approximately 5.7 × 10^−8^ cm^2^·s^−1^ [261]. Furthermore, the intercalation of PA molecules could enhance the hydrophobicity of the material in aqueous electrolyte, effectively suppressing the decomposition and dissolution of VOPO_4_·2H_2_O [261]. Wu et al. demonstrated a strategy involving the topochemical incorporation of Zn^2+^ ions into VOPO_4_·2H_2_O to form Zn_0.4_VOPO_4_·0.8H_2_O (Figure 14c) [262]. This approach enables the stabilization of the layered framework without altering the intrinsic in‐plane atomic arrangement or the local coordination environment. Ling et al. proposed an in situ transformation strategy to fabricate vertically aligned VOPO_4_·2H_2_O nanosheets with oxygen vacancies (VO‐d‐VOPO_4_), which exhibited (200) preferential orientation [263]. The abundantly exposed (200) planes provide tetragonal channels that facilitate fast in‐layer and cross‐layer Zn^2+^ migration (Figure 14d), thereby enhancing ion transfer kinetics and reversible capacity [263]. Besides, the VO‐d‐VOPO_4_ electrode delivers an exceptional discharge capacity (213.5 mAh g^−1^), impressive rate capability, and remarkable long‐term cycling stability [264]. By pre‐intercalating and polymerizing aniline within VOPO_4_ layers (Figure 14e), Olabintan et al. created a conductive hybrid cathode (PA‐VP) [265]. This design utilizes the π‐conjugated PANI as a multifunctional pillar that screens electrostatic interactions to facilitate Zn^2+^ insertion, promotes electron transport via vanadium valence modulation, and creates a favorable microstructure for ion access, collectively enhancing the material's properties [264]. In brief, research on VOPO_4_ cathodes for AZIBs is aimed at overcoming inherent limitations in ion kinetics and structural stability. Promising solutions span interlayer spacing expansion through molecular pillaring, Zn^2+^ pre‐stabilization, nanostructuring with oxygen vacancies, and the integration of conductive polymers to collectively boost electrochemical performance.

LDHs, representative 2D materials, have achieved remarkable progress in energy conversion and storage, demonstrating unique strengths and broad prospects for AZIB cathode development [204, 205, 206, 207, 208, 209]. The intrinsic layered architecture of LDHs endows them with abundant active sites and favorable ion‐exchange capability, delivering high theoretical specific capacity and good structural adjustability. Unfortunately, conventional LDHs face major obstacles in practical applications: strong intralayer coordination bonds degrade electronic conductivity, and anisotropic ion transport impedes reaction kinetics [203, 204, 205, 207]. To overcome these limitations, researchers have developed various interfacial engineering strategies. For instance, Wei et al. employed an electrochemical cycling activation (ECA) approach to in situ reconstruct NiCo‐LDHs into a heterostructured architecture, thereby activating additional active sites for Zn^2+^ storage and accelerating ion diffusion [204]. The optimized ECA‐NiCo‐LDHs cathode delivered a high specific capacity of 181.5 mAh g^−1^ at a current density of 1 A g^−1^ and retained 75% of its initial capacity after 700 cycles at 5 A g^−1^. In another study, Wei et al. constructed heterojunctions with different work functions to generate a strong built‐in electric field, which drives interfacial charge redistribution, enhances electron transport kinetics, and optimizes ion adsorption behavior [205]. The resulting BS@CN‐LDH electrode achieved a high specific capacity of 450.5 mAh g^−1^ at a current density of 2 mA cm^−2^. Furthermore, Li et al. adopted a redox‐driven in situ etching strategy to fabricate strongly coupled heterojunctions encapsulated within a carbon matrix. The 3D conductive network ensures fast electron transfer and superior structural durability [207]. The optimized electrode delivered a specific capacity of 235.6 mAh g^−1^ at 1 A g^−1^ and maintained 91.7% of its initial capacity even at a high current density of 10 A g^−1^. Such interfacial engineering tactics enhance electrochemical performance through synergistic multi‐scale mechanisms. Heterogeneous interfaces enrich active sites, built‐in electric fields promote electron migration, carbon confinement mitigates volume expansion, and interfacial strain generates lattice defects and enlarges interlayer spacing. Equipped with these advantages, interfacially engineered 2D LDHs serve as high‐performance AZIB cathodes. This work sheds new light on the rational design of low‐cost, eco‐friendly next‐generation energy storage systems.

MXenes have emerged as a class of promising cathode materials for AZIBs [41, 51, 52]. A mainstream research direction is to exploit MXenes as highly conductive scaffolds and functional substrates. Hybridizing MXenes with high‐capacity active materials with unsatisfactory cycling performance enables the construction of composite cathodes, addressing the intrinsic limitations of pristine MXenes including low specific capacity and working voltage [208, 209, 210, 211]. Within these heterostructures, MXenes endow the composites with boosted electronic and ionic conductivity, enlarged interlayer spacing, abundant redox‐active centers, and robust structural stability. These favorable characteristics guarantee fast and reversible Zn^2+^ (de)intercalation. As a representative work, Zhao et al. engineered flexible self‐standing thin‐film electrodes based on 6CN‐HAT and MXene nanosheets [208]. The incorporation of MXene optimized electrical conductivity and structural integrity, while creating more accessible active sites. In another study, Zeng et al. pioneered a binder‐free self‐standing film cathode [209]. This ternary composite combines the advantages of different components: MXene delivers high conductivity and sufficient Zn^2+^ storage sites, single‐walled carbon nanotubes enhance mechanical strength and mitigate charge‐transfer resistance, and α‐MnO_2_ serves as the dominant active material for capacity contribution. The resultant composite delivers far better specific capacity, rate capability, and cyclic stability than its single counterparts. Beyond that, interfacial coupling between MXene and active materials modulates local electronic configurations, lowers the Zn^2+^ diffusion barrier, and optimizes electrochemical reaction kinetics. Zhang et al. synthesized highly crystalline CoHCF nanotube/MXene composites with minimized structural defects and coordinated water content [210]. Combined XPS characterization and DFT simulations demonstrate the formation of Co–C covalent bonds at the heterointerface. Such interfacial bonding accelerates electron migration and rigidifies the crystal framework, avoiding structural deterioration during prolonged charge–discharge processes. In short, the design of MXene‐based composite cathodes offers an effective route toward advanced AZIBs that integrate high energy density, high power density, and excellent long‐cycle stability. The electrochemical performances of various 2D layered cathode materials for AZIBs are systematically summarized in Table 2.

**TABLE 2:** Summary of performances of 2D layered materials as the cathode for AZIBs.

Electrode material	Electrolyte	Rate performance	Discharge capability	Cycle capability	Ref.
Cr_2_V_4_O_13_/V_2_O_5_‐2	3 M Zn(CF_3_SO_3_)_2_	40% retention (2 A g^−1^)	378 mAh g^−1^ (0.1 A g^−1^)	1000 cycles, 86% retention (1 A g^−1^)	[265]
V_2_O_5_‐40	3 M Zn(CF_3_SO_3_)_2_	52% retention (4 A g^−1^)	418 mAh g^−1^ (0.1 A g^−1^)	1200 cycles, 77% retention (4 A g^−1^)	[266]
V_2_O5/V_6_O_13_ heterostructure	2 M ZnSO_4_	29.6% retention (5 A g^−1^)	338 mAh g^−1^ (0.1 A g^−1^)	4000 cycles, 97% retention (5 A g^−1^)	[267]
K_0.48_6V_2_O_5_	3 M Zn(CF_3_SO_3_)_2_	40% retention (3.2 A g^−1^)	339 mAh g^−1^ (0.05 A g^−1^)	1400 cycles, 74.8% retention (3 A g^−1^)	[268]
PVO‐60	3 M Zn(CF_3_SO_3_)_2_	52.5% retention (20 A g^−1^)	375 mAh g^−1^ (1 A g^−1^)	1000 cycles, 95% retention (2 A g^−1^)	[269]
V_2_O_5_ nanobelts/rGO	3 M ZnSO_4_	64.6% retention (1 A g^−1^)	175 mAh g^−1^ (0.1 A g^−1^)	200 cycles, 77% retention (0.1 A g^−1^)	[270]
V_6_O_13_‐Vo‐N	3 M Zn(CF_3_SO_3_)_2_	38.4% retention (10 A g^−1^)	431 mAh g^−1^ (0.1 A g^−1^)	10000 cycles, 90% retention (10 A g^−1^)	[271]
NVO	2 M ZnSO_4_	59.6% retention (10 A g^−1^)	339 mAh g^−1^ (0.1 A g^−1^)	1000 cycles, 98% retention (1 A g^−1^)	[272]
K_2_V_6_O_16_·nH_2_O	2.8 M Zn(CF_3_SO_3_)_2_	47% retention (15 A g^−1^)	263mAh g^−1^ (0.5 A g^−1^)	5000 cycles, 97% retention (10 A g^−1^)	[273]
INVO‐2	3 M Zn(CF_3_SO_3_)_2_	33.5% retention (20 A g^−1^)	464mAh g^−1^ (0.2 A g^−1^)	5000 cycles, 86% retention (10 A g^−1^)	[274]
ZSO–20 AHA	2 M ZnSO_4_	—	403 mAh g^−1^ (0.2 A g^−1^)	700 cycles, 82% retention (5 A g^−1^)	[275]
Au‐V_6_O_13_	3 M Zn(CF_3_SO_3_)_2_	37.7% retention (10 A g^−1^)	330.4 mAh g^−1^ (0.5 A g^−1^)	1445 cycles, 93% retention (10 A g^−1^)	[276]
V/O‐NHVO	3 M Zn(CF_3_SO_3_)_2_	45.8% retention (5 A g^−1^)	545 mAh g^−1^ (0.1 A g^−1^)	2000 cycles, 87.9% retention (5 A g^−1^)	[277]
NVO‐Cl	2 M Zn(CF_3_SO_3_)_2_	101% retention (0.2 A g^−1^)	500 mAh g^−1^ (0.1 A g^−1^)	7000 cycles, 109% retention (4 A g^−1^)	[278]
P_1.0_VO	3 M Zn(CF_3_SO_3_)_2_	96.9% retention (0.1 A g^−1^)	481 mAh g^−1^ (0.1 A g^−1^)	1000 cycles, 86.6% retention (1 A g^−1^)	[279]
V_2_O_5_/Zn	2 M ZnSO_4_	31.4% retention (10 A g^−1^)	66.3 mAh g^−1^ (0.05 A g^−1^)	20000 cycles, 83% retention (10 A g^−1^)	[280]
Co‐NVO	3 M Zn(CF_3_SO_3_)_2_	36.7% retention (8 A g^−1^)	394 mAh g^−1^ (0.1 A g^−1^)	3000 cycles, 92.1% retention (10 A g^−1^)	[281]
p‐HVO_x_@C‐700	2 M Zn(CF_3_SO_3_)_2_	85% retention (10 A g^−1^)	464 mAh g^−1^ (0.2 A g^−1^)	2500 cycles, 89.3% retention (10 A g^−1^)	[282]
Co‐δ‐MnO_2_	2 M ZnSO_4_ and 0.25 M MnSO_4_	56.9% retention (3 A g^−1^)	409mAh g^−1^ (0.1 A g^−1^)	1000 cycles, 77.8% retention (1 A g^−1^)	[283]
H‐MnO_2_‐x‐3	2 M ZnSO_4_ and 0.1 M MnSO_4_	18.0% retention (3 A g^−1^)	401mAh g^−1^ (0.1 A g^−1^)	1000 cycles, 73% retention (3 A g^−1^)	[284]
HMS	2 M ZnSO_4_ and 0.2 M MnSO_4_	63.2% retention (2 A g^−1^)	375 mAh g^−1^ (0.1 A g^−1^)	1000 cycles, 84% retention (2 A g^−1^)	[285]
δ‐MnO_2_‐1.5 h/Gr	3.0 M ZnSO_4_ and 0.1 M MnSO_4_	35.9% retention (2 A g^−1^)	301 mAh g^−1^ (0.1 A g^−1^)	1000 cycles, 85.8% retention (1 A g^−1^)	[286]
MA‐MnO	2 M ZnSO_4_ and 0.2 M MnSO_4_	80% retention (1 A g^−1^)	500 mAh g^−1^ (1 A g^−1^)	1350 cycles, 150% retention (10 A g^−1^)	[287]
P–MnO_2_	2 M ZnSO_4_	39.9% retention (5 A g^−1^)	368 mAh g^−1^ (0.1 A g^−1^)	1100 cycles, 74% retention (1 A g^−1^)	[288]
MnO_2_@CQDs	2 M ZnSO_4_ and 0.2 M MnSO_4_	37.3% retention (2 A g^−1^)	193 mAh g^−1^ (0.1 A g^−1^)	500 cycles, 90% retention (1 A g^−1^)	[289]
2%Ni‐MnO_2_/PPy	2 M ZnSO_4_	38% retention (1 A g^−1^)	511 mAh g^−1^ (0.1 A g^−1^)	600 cycles, 45% retention (1 A g^−1^)	[290]
MnO_2_/MXene_1:0.5_/CF	2 M ZnSO_4_ and 0.2 M MnSO_4_	21.5% retention (1 A g^−1^)	464mAh g^−1^ (0.1 A g^−1^)	1000 cycles, 76.4% retention (2 A g^−1^)	[291]
MXene@Mn_3_O_4_@PPy	2 M ZnSO_4_	34.9% retention (5 A g^−1^)	424 mAh g^−1^ (0.1 A g^−1^)	9000 cycles, 81% retention (5 A g^−1^)	[292]
Cu_2_‐KMO	2 M Zn (CF_3_SO_3_)_2_ and 0.2 M MnSO_4_	36.6% retention (2 A g^−1^)	336 mAh g^−1^ (0.1 A g^−1^)	1000 cycles, 75% retention (1 A g^−1^)	[293]
MnO_2_ (rGO)	2.4 M Zn(CF_3_SO_3_)_2_ and 0.1 M MnTFSI	46% retention (3 A g^−1^)	373 mAh g^−1^ (0.03 A g^−1^)	2000 cycles, 35% retention (3 A g^−1^)	[238]
MON‐coated MnO_2_	2 M ZnSO_4_	36% retention (20 A g^−1^)	209 mAh g^−1^ (0.2 A g^−1^)	1000 cycles, 85.9% retention (10 A g^−1^)	[294]
MnO/C	2 M Zn(CF_3_SO_3_)_2_	48.% retention (1 A g^−1^)	96.4 mAh g^−1^ (0.1 A g^−1^)	10000 cycles, 73.8% retention (1 A g^−1^)	[295]
MnO@N‐C‐500	2 M ZnSO_4_	26.5% retention (2 A g^−1^)	250 mAh g^−1^ (0.1 A g^−1^)	200 cycles, 103% retention (0.5 A g^−1^)	[296]
MoS_2_–Se	3 M Zn(CF_3_SO_3_)_2_	44.9% retention (10 A g^−1^)	109 mAh g^−1^ (1 A g^−1^)	500 cycles, 93% retention (4 A g^−1^)	[297]
PPy–MoS_2_	3 M Zn(CF_3_SO_3_)_2_	41% retention (10 A g^−1^)	404 mAh g^−1^ (1 A g^−1^)	1000 cycles, 83.7% retention (5 A g^−1^)	[298]
V_2_O_5_@MoS_2_	3 M Zn(CF_3_SO_3_)_2_	—	325 mAh g^−1^ (1 A g^−1^)	6000 cycles, 56% retention (10 A g^−1^)	[299]
MoS_2_‐PEG	3 M Zn(CF_3_SO_3_)_2_	63.3% retention (10 A g^−1^)	139 mAh g^−1^ (0.1 A g^−1^)	115 cycles, 84% retention (1 A g^−1^)	[300]
F/P‐MoS_2_	3 M Zn(CF_3_SO_3_)_2_	63.6% retention (2 A g^−1^)	140mAh g^−1^ (0.1 A g^−1^)	200cycles, 98% retention (1 A g^−1^)	[301]
MoS_1.8_Se_0.2_/rGO	3 M Zn(CF_3_SO_3_)_2_	29.1% retention (8 A g^−1^)	214 mAh g^−1^ (0.1 A g^−1^)	1000cycles, 52.4% retention (1 A g^−1^)	[302]
MoS_2_/MWCNTs	2 M ZnSO_4_	62.4% retention (3 A g^−1^)	218 mAh g^−1^ (0.1 A g^−1^)	1000cycles, 74% retention (1 A g^−1^)	[303]
MoS_2_/PANI	3 M Zn(CF_3_SO_3_)_2_	45.8% retention (2 A g^−1^)	182 mAh g^−1^ (0.1 A g^−1^)	1000cycles, 86% retention (1 A g^−1^)	[304]
MoS_2_‐nH_2_O	3 M Zn(CF_3_SO_3_)_2_	38.8% retention (3 A g^−1^)	165 mAh g^−1^ (0.1 A g^−1^)	800cycles, 88% retention (2 A g^−1^)	[305]
1T MoS_2_	3 M Zn(CF_3_SO_3_)_2_	47.4% retention (10 A g^−1^)	191 mAh g^−1^ (0.1 A g^−1^)	400cycles, 98% retention (5 A g^−1^)	[306]
MoS_2‐x_	3 M Zn(CF_3_SO_3_)_2_	58.3% retention (10 A g^−1^)	138 mAh g^−1^ (0.1 A g^−1^)	1000cycles, 88% retention (2 A g^−1^)	[307]
MoS_2_/Ti_3_C_2_T_x_	3 M Zn(CF_3_SO_3_)_2_	38.3% retention (10 A g^−1^)	277 mAh g^−1^ (0.1 A g^−1^)	_1000cycles, 86% retention (1 A g‐1)	[308]
MoS_2_@CNTs	3 M Zn(CF_3_SO_3_)_2_	51.7% retention (3 A g^−1^)	180 mAh g^−1^ (0.1 A g^−1^)	500cycles, 80% retention (1 A g^−1^)	[309]
Al2‐VO_2_	3 M Zn(CF_3_SO_3_)_2_	42.9% retention (10 A g^−1^)	452 mAh g^−1^ (0.1 A g^−1^)	10000cycles, 70% retention (1 A g^−1^)	[310]
POMA‐V_2_O_5_	3 M Zn(CF_3_SO_3_)_2_	33.4% retention (20 A g^−1^)	401 mAh g^−1^ (0.2 A g^−1^)	10000cycles, 68% retention (10 A g^−1^)	[311]
VS‐450	3 M Zn(CF_3_SO_3_)_2_	29.4% retention (3 A g^−1^)	372 mAh g^−1^ (0.1 A g^−1^)	5000cycles, 93% retention (2 A g^−1^)	[312]
Al_0.060_V_2_O_5_	3 M ZnSO_4_	—	358 mAh g^−1^ (1 A g^−1^)	100cycles, 89% retention (1 A g^−1^)	[313]

## 4 2D Layered Materials in Separators and Electrolytes

**FIGURE 15:**
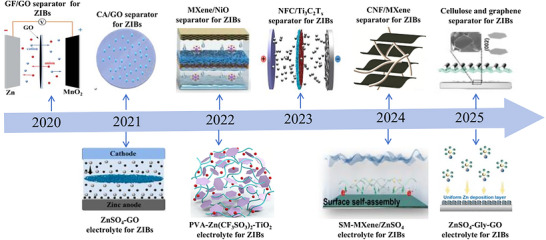
Timeline of the development of 2D layered materials as separators and electrolytes in AZIBs. From left to right: Reproduced with permission [314]. Copyright 2020, the Royal Society of Chemistry. Reproduced with permission [315]. Copyright 2021, American Chemical Society. Reproduced with permission [316]. Copyright 2022, American Chemical Society. Reproduced with permission [317]. Copyright 2023, Elsevier. Reproduced with permission [318]. Copyright 2024, Elsevier. Reproduced with permission [319]. Copyright 2025, Elsevier. Reproduced with permission [320]. Copyright 2021, American Chemical Society. Reproduced with permission [321]. Copyright 2022, Elsevier. Reproduced with permission [322]. Copyright 2024, Elsevier. Reproduced with permission [225]. Copyright 2025, the Royal Society of Chemistry.

The integration of 2D layered materials (e.g., graphene, MXenes, hexagonal boron nitride (h‐BN), and TMDs) into battery separators and electrolytes represents a cutting‐edge strategy to address key limitations in AZIBs. Figure 15 illustrates the timeline of the development of 2D layered materials as separators and electrolytes in ZIBs [314, 315, 316, 317, 318, 319, 320, 321, 322, 323]. These materials offer a distinctive set of physicochemical characteristics of ultra‐high aspect ratio, outstanding mechanical robustness, chemically tunable surfaces, and exceptional thermal and electrochemical stability. By leveraging these properties, researchers can engineer multifunctional components that not only address the inherent limitations of existing battery materials but also open pathways to significantly enhanced safety, longevity, and energy density. Table 3 comprehensively summarizes the electrochemical performances of 2D layered materials as separators and electrolytes for AZIBs.

**TABLE 3:** Summary of the performances of 2D layered materials as separators and electrolytes for AZIBs.

Material	Electrolyte	Rate performance	Discharge capability	Cycle capability	Ref.
MCNF separator	2 M ZnSO_4_	70.98% retention (2 A g^−1^)	355 mAh g^−1^ (0.1 A g^−1^)	1800 cycles, 112.6% retention (2 A g^−1^)	[356]
GF@NCF700@GF	2 M ZnSO_4_	—	277.4 mAh g^−1^ (1 A g^−1^)	2200 cycles, 89% retention (3 A g^−1^)	[357]
Janus separator	2 M ZnSO_4_	56.27% retention (10 A g^−1^)	231 mAh g^−1^ (0.5 A g^−1^)	1000 cycles, 75% retention (5 A g^−1^)	[358]
CG Separator	2 M Zn(CF_3_SO_3_)_2_	33% retention (1 A g^−1^)	232 mAh g^−1^ (0.1 A g^−1^)	800 cycles, 95.3% retention (1 A g^−1^)	[359]
GF/GO1	2 M ZnSO_4_	41% retention (1 A g^−1^)	117 mAh g^−1^ (0.2 A g^−1^)	500cycles, 72.94% retention (0.5 A g^−1^)	[360]
Pc‐GF	2 M ZnSO_4_	67% retention (1 A g^−1^)	152.3 mAh g^−1^ (0.2 A g^−1^)	6000 cycles, 88.42% retention (1 A g^−1^)	[361]
SPAES@GF	2 M ZnSO_4_	77.94% retention (2 A g^−1^)	264.2 mAh g^−1^ (0.1 A g^−1^)	600 cycles, 60% retention (1 A g^−1^)	[362]
10%ZIF‐8/MXene/PAN/PU	2 M Zn(CF_3_SO_3_)_2_	—	37.77 mAh g^−1^ (5 A g^−1^)	500 cycles, 73.33% retention (1 A g^−1^)	[363]
Ag/MXene	2 M ZnSO_4_	58.32% retention (5 A g^−1^)	95.26 mAh g^−1^ (0.2 A g^−1^)	10000 cycles, 90.67% retention (0.5 A g^−1^)	[364]
CM‐15 separator	2 M ZnSO_4_ and 0.2 M MnSO_4_	63.45% retention (2 A g^−1^)	354.9 mAh g^−1^ (0.1 A g^−1^)	200 cycles, 92.2% retention (1 A g^−1^)	[365]
Ti_3_C_2_T_x_ MXene	2 M ZnSO_4_ and 0.2 M MnSO_4_	—	165 mAh g^−1^ (0.5 A g^−1^)	500 cycles, 85.6% retention (1 A g^−1^)	[339]
Ti_3_C_2_‐Wrapped PP Separator	2 M ZnSO_4_	34.52% retention (30 A g^−1^)	350 mAh g^−1^ (0.5 A g^−1^)	3000 cycles, 83% retention (10 A g^−1^)	[366]
C_3_N_4_@GF	2 M ZnSO_4_	35.93% retention (2 A g^−1^)	320 mAh g^−1^ (0.2 A g^−1^)	500 cycles, 99% retention (2 A g^−1^)	[367]
3%HT	2 M ZnSO_4_	59.37% retention (4 A g^−1^)	480 mAh g^−1^ (0.5 A g^−1^)	2000 cycles, 85% retention (0.5 A g^−1^)	[368]
GO electrolyte additive	2 M ZnSO_4_	76% retention (5 A g^−1^)	75 mAh g^−1^ (2 A g^−1^)	250 cycles, 93% retention (5 A g^−1^)	[369]
rGO electrode	2 M ZnSO_4_	42.3% retention (0.005 A g^−1^)	233.7 mAh g^−1^ (0.005 A g^−1^)	2000 cycles, 90.9% retention (0.005 A g^−1^)	[370]
GOQDs‐modified electrolyte	3 M Zn(CF_3_SO_3_)_2_	—	132.79 mAh g^−1^ (1 A g^−1^)	3000 cycles, 81% retention (1 A g^−1^)	[371]
ZS/Q2‐0.7 electrolyte	2 M ZnSO_4_	41.58% retention (5 A g^−1^)	344.19mAh g^−1^ (0.1 A g^−1^)	25000 cycles, 85.48% retention (5 A g^−1^)	[372]
ZS‐NF0.6 electrolyte	2 M ZnSO_4_	51.42% retention (5 A g^−1^)	374.5mAh g^−1^ (0.2 A g^−1^)	1000 cycles, 36% retention (5 A g^−1^)	[373]
FCDs additive	2 M ZnSO_4_ and 0.2 M MnSO_4_	—	—	2000 cycles, 81.4% retention (3 A g^−1^)	[374]
FCQDs	2 M ZnSO_4_	—	103.9 mAh g^−1^ (1 A g^−1^)	600 cycles, 70.8% retention (0.2 A g^−1^)	[350]
QDP	2 M ZnSO_4_	3.4% retention (5 A g^−1^)	106.9mAh g^−1^ (0.1 A g^−1^)	1000 cycles, 73% retention (0.5 A g^−1^)	[375]
CQDs	2 M ZnSO_4_	—	265mAh g^−1^ (0.1 A g^−1^)	300 cycles, 74% retention (0.2 A g^−1^)	[376]
SGQDs	1 M ZnSO_4_	—	105mAh g^−1^ (5 A g^−1^)	1500 cycles, 81% retention (5 A g^−1^)	[377]

### 4.1 2D Layered Materials in Separators

The separator is an indispensable component that plays a key role in determining the performance of electrochemical devices. An ideal separator should possess high ionic conductivity but low electronic conductivity, robust mechanical properties, and a high affinity for the Zn anode [324, 325, 326, 327]. The commercial glass fiber (GF) separator is widely used in aqueous ZIBs due to its excellent chemical stability, high porosity, and superior wettability with aqueous electrolytes [328, 329, 330, 331]. It ensures intimate contact with both electrodes, and the interface conditions, particularly on the anode side, critically influence battery performance. However, its large, uneven pores promote unstable Zn deposition and dendrite penetration. Besides, the separator is mechanically fragile (tensile strength: 0.25–0.5 MPa) and thick (∼300 µm), which compromises durability and volumetric energy density [332, 333, 334]. Notably, the surface functionality of separators is pivotal for ZIB stability, governing interfacial chemistry at both electrodes. Advances in the research of functionalized separators are paving the way for the efficient preparation of separators using innovative 2D materials (e.g., rGO, MXenes) [335, 336, 337]. Coating offers a direct method to engineer these properties and is universally applicable to commercial or synthesized separators. For example, Luo et al. developed an ultrathin GO‐modified cellulose acetate (CA) separator (4 µg cm^−2^) using a simple filtration method [315]. SEM characterization confirms that zinc deposited on the GO/CA separator forms compact, homogeneous flakes that grow in parallel with the substrate. The GO nanosheets facilitate uniform Zn nucleation and epitaxial (002) deposition, thereby yielding a dendrite‐free anode [315]. Wu et al. proposed a regenerated cellulose/graphene composite separator to stabilize the Zn anode and prolong ZIB lifespan [319]. As evidenced by SEM characterization, electrodeposited zinc with the composite separator adopts a compact, well‐distributed hexagonal platelet morphology, and these platelets propagate in a direction parallel to the substrate. On the other hand, the GF separator induces uneven zinc deposition, resulting in a rough surface populated by sharp dendrites and disordered spherical agglomerates. The cellulose component regulates Zn^2+^ solvation and kinetics, while graphene guides (002)‐oriented deposition via a low lattice mismatch interface [319]. This hybrid design also yields a high tensile strength of 91.23 MPa, surpassing previous records and providing critical protection against short circuits from dendrites or external forces.

**FIGURE 16:**
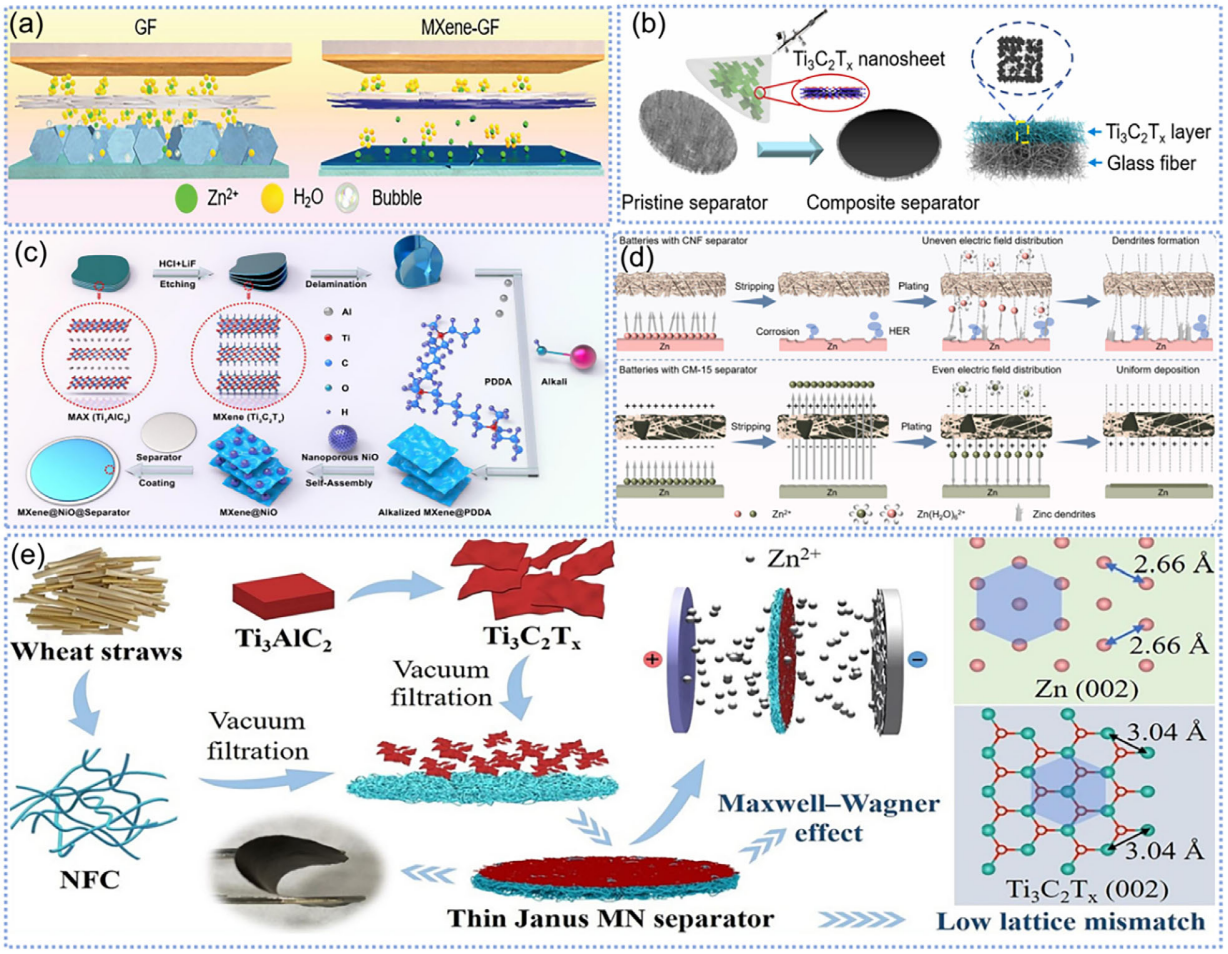
(a) Schematic diagram of AZIB equipped with GF and MXene‐GF as separators [338]. Copyright 2022, Wiley‐VCH GmbH. (b) Schematic illustration of the Ti_3_C_2_T_x_@glass fiber composite separator for stabilizing the Zn anode [339]. Copyright 2023, Elsevier. (c) Schematic illustration of the MXene@NiO modified separator synthesis [316]. Copyright 2022, American Chemical Society. (d) Schematic diagram illustrating the Zn^2+^ transport mechanism through the CNF and CM‐15 separators [328]. Copyright 2024, Elsevier. (e) Schematic of the preparation and function of the MN separator [317]. Copyright 2023, Elsevier.

MXenes, a class of 2D transition metal carbides, nitrides, and carbonitrides, are ideal candidates due to their high conductivity, large surface area, tunable hydrophilicity, rich surface chemistry, and excellent mechanical properties. MXene's high metal‐like conductivity and zincophilic nature make it an ideal host for guiding initial nucleation and ensuring homogeneous zinc deposition [316, 317, 318]. Its unique hexagonal lattice also provides a high lattice match with zinc, favoring planar growth. These properties collectively enable MXene to redistribute Zn^2+^ flux, balance the interfacial electric field, and lower desolvation energy. For example, Su et al. designed a scalable Janus separator by spray‐printing Ti_3_C_2_T_x_ MXene onto one side of a GF [318]. The resulting MXene‐GF separator features abundant polar groups, good wettability, and high ionic conductivity, thereby homogenizing current distribution and improving Zn nucleation (Figure 16a) [338]. Moreover, its adjustable dielectric constant (optimized at 53.5) generates a directional electric field. This field expedites Zn^2+^ migration and excludes anions, enabling dendrite‐free plating in symmetric cells for 1180 h at 1 mA cm^−2^ [338]. Zhou et al. developed an ion‐sieving Janus separator by spray‐coating Ti_3_C_2_T_x_ MXene onto a glass fiber (Figure 16b) [339]. The MXene's properties enable the separator to homogenize the Zn anode's electric field and guide uniform deposition. Simultaneously, its inherent electronegativity selectively blocks H^+^ and SO_4_^2−^ ions, significantly suppressing hydrogen evolution and side reactions at the anode [339]. An et al. developed a freestanding, zincophilic MXene/nanoporous oxide separator to stabilize Zn anodes (Figure 16c) [316]. The oxide framework offers high porosity and surface area. Integrated with MXene, the heterostructure homogenizes the electric field and ion flux, accelerates diffusion, and lowers current density, collectively ensuring uniform Zn deposition and suppressed side reactions [218]. Huang et al. developed an ultrathin separator (denoted as CM‐15) composed of cellulose nanofibers and MXene (Figure 16d) [318]. The MXene component provides strong zincophilicity and high electrical/thermal conductivity, functioning as an ion pump to accelerate Zn^2+^ transport [318]. This mechanism significantly increases the Zn^2+^ transference number, promoting the deposition of homogeneous nuclei and yielding a dendrite‐free Zn anode. Wu et al. reported a Janus separator composed of a Ti_3_C_2_T_x_ layer on nanocellulose film (Figure 16e), exhibiting superior thinness (32 µm) and tensile strength (215.3 MPa) [317]. Moreover, the Ti_3_C_2_T_x_ layer improves ion transport and reaction kinetics, lowers the desolvation energy and nucleation overpotential, thereby effectively inhibiting dendrites and side reactions [317]. Overall, an ultrathin CF/MXene separator is a promising strategy for achieving rapid ion transport, homogeneous deposition, and improved electrochemical properties in AZIBs.

### 4.2 2D Layered Materials in Electrolytes

**FIGURE 17:**
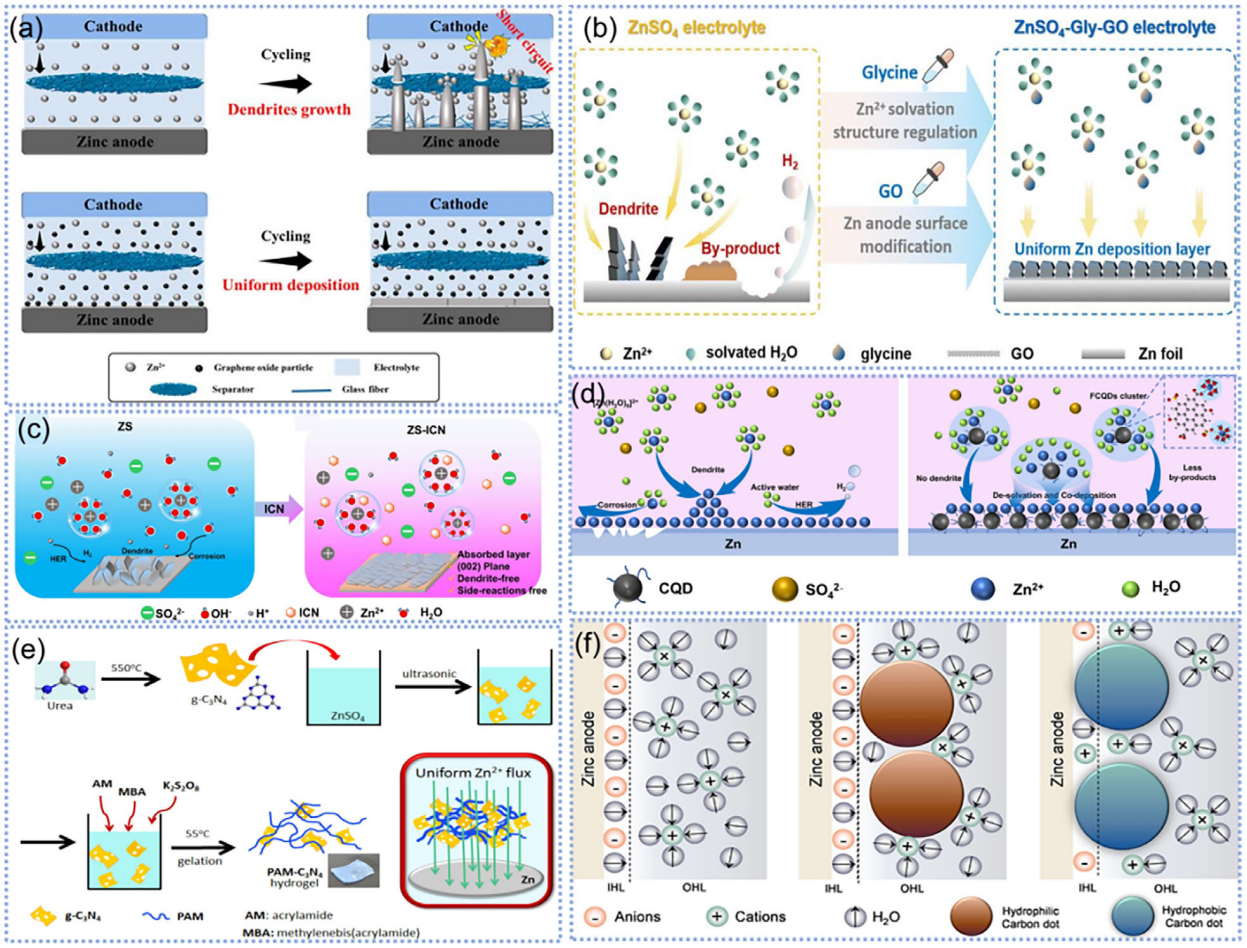
(a) Schematic diagram of AZIB with GO additive vs. without additive [320]. Copyright 2021, American Chemical Society. (b) Schematic diagram of a stable Zn anode enabled by dual additives [323]. Copyright 2025, the Royal Society of Chemistry. (c) Schematic illustration of the Zn deposition process with ICN additive vs. without additive [349]. Copyright 2024, Elsevier. (d) Schematic diagrams of Zn deposition in a 2 M ZnSO_4_ electrolyte vs. a composite electrolyte with FCQDs [350]. Copyright 2024, Elsevier. (e) Schematic illustration of the preparation process for the PAM‐C_3_N_4_ composite hydrogel [351]. Copyright 2025, Elsevier. (f) Schematic illustration comparing the Helmholtz layer structures on Zn anodes in pristine ZnSO_4_ electrolyte, ZnSO_4_ with hydrophilic CDs, and ZnSO_4_ with hydrophobic CDs [352]. Copyright 2025, American Chemical Society.

Electrolyte optimization using functional additives is a practically promising strategy to regulate Zn^2+^ plating/stripping and simultaneously mitigate interfacial side reactions [340, 341, 342, 343]. Typically, such additives can regulate zinc deposition by adsorbing at the electrode‐electrolyte interface or by participating in the Zn^2+^ solvation structure. These mechanisms promote uniform zinc nucleation and suppress side reactions. Notably, the introduction of functional 2D nanosheets as electrolyte additives presents a dynamic strategy for stabilizing metal anodes [344,–346]. These nanosheets act as intelligent, mobile modifiers that preferentially adsorb onto localized protrusions on the anode surface due to their higher surface energy. Upon adsorption, they function as nano‐scale current regulators, mitigating the ‘tip effect’ and guiding a more uniform ion flux [347, 348, 349, 350, 353, 354, 355]. This capability enables the system to autonomously rectify initial inhomogeneities, resulting in a continuous self‐healing mechanism that maintains an exceptionally smooth deposition interface and effectively suppresses dendritic growth throughout cycling. For example, Abdulla et al. developed a hybrid electrolyte incorporating GO as a functional additive [320]. The GO additive promotes a uniform electric field distribution and reduces the Zn^2+^ nucleation overpotential (Figure 17a), which enabled smooth zinc electrodeposition and enhanced reaction kinetics [320]. 3D laser scanning images clearly show that after cycling, the zinc anode without GO exhibits numerous red protrusions (indicating dendrites), whereas the GO‐containing zinc anode surface remains smooth and flat. Electric field distribution simulations reveal that in the GO‐containing system, GO adsorbs onto the zinc surface, enabling a uniform electric field distribution and thereby fundamentally eliminating the tip effect. The zinc symmetric battery with graphene oxide additive delivers stable cycling over 650 h at 1 mA cm^−2^ and sustains 140 h even at 10 mA c^−2^, showcasing excellent rate adaptability and cycling durability [320]. To enhance Zn anode performance, Wan et al. incorporated glycine and GO as dual additives into the ZnSO_4_ electrolyte [323]. They found that glycine modulates the Zn^2^^+^ solvation structure, weakening the Zn^2+^‐SO_4_^2−^ interaction and thus inhibiting HER and sulfate byproducts (Figure 17b) [323]. Meanwhile, GO adsorbs on the electrode to form a functional rGO layer that serves as both a protective interface and a nucleation substrate, while also facilitating charge transfer and uniform Zn deposition for a homogeneous electric field [323]. Zhang et al. demonstrated a highly reversible Zn anode by employing atomic‐scale inorganic carbon nanomaterials (ICN) as a multifunctional electrolyte additive in a conventional ZnSO_4_ solution (Figure 17c) [349]. The ICN modulates the Zn^2+^ solvation structure to suppress side reactions while forming a protective passivation layer on the anode [349]. Its low lattice mismatch with Zn further guides preferential (002) deposition, yielding a dense, smooth morphology that fundamentally prevents dendrites and corrosion [349]. Wang et al. proposed a one‐pot hydrothermal strategy to create a functionalized carbon quantum dots (FCQDs)/ZnSO_4_ hybrid electrolyte from 0.01 M glucose, effectively suppressing dendrites and side reactions (Figure 17d) [350]. The modified solvation sheath lowers the de‐solvation energy barrier, which collectively contributes to inhibiting water‐induced parasitic reactions and stabilizing the Zn anode [350]. Wang et al. developed a polyacrylamide (PAM)‐C_3_N_4_ composite hydrogel electrolyte by incorporating 2D g‐C_3_N_4_ nanoplates into the PAM matrix (Figure 17e) [351]. The porous g‐C_3_N_4_ nanosheets function dually to homogenize Zn^2+^ flux and accelerate zinc‐ion dehydration kinetics, consequently enabling uniform Zn deposition and suppressed dendrite growth [351]. Temperature‐dependent EIS measurements show that the activation energy for Zn^2+^ deposition/stripping in the PAM‐C_3_N_4_ electrolyte is 53.87 kJ mol^−1^, significantly lower than that of the pure PAM electrolyte (58.65 kJ mol^−1^). This lower activation energy directly demonstrates faster desolvation kinetics. The integration of 2D g‐C_3_N_4_ nanosheets thus presents an effective strategy for mitigating dendrite formation and enhancing the overall performance of zinc‐ion batteries [351]. Yang et al. engineered a dynamic interface featuring a monolayer of hydrophobic carbon dots (CDs) to simultaneously regulate both the inner and outer Helmholtz layers (Figure 17f) [352]. This synergistic effect ensured the dynamic and reversible behavior of the CD monolayer during repeated plating/stripping, thus achieving long‐term cycling stability without depleting the trace CD additive [352]. In short, electrolyte optimization with various functional additives is a practical strategy for achieving highly reversible zinc anodes. These additives function by modulating the electrode‐electrolyte interface and the Zn^2+^ solvation structure, thereby promoting uniform zinc deposition and suppressing side reactions.

## 5 Summary and Outlook

**FIGURE 18:**
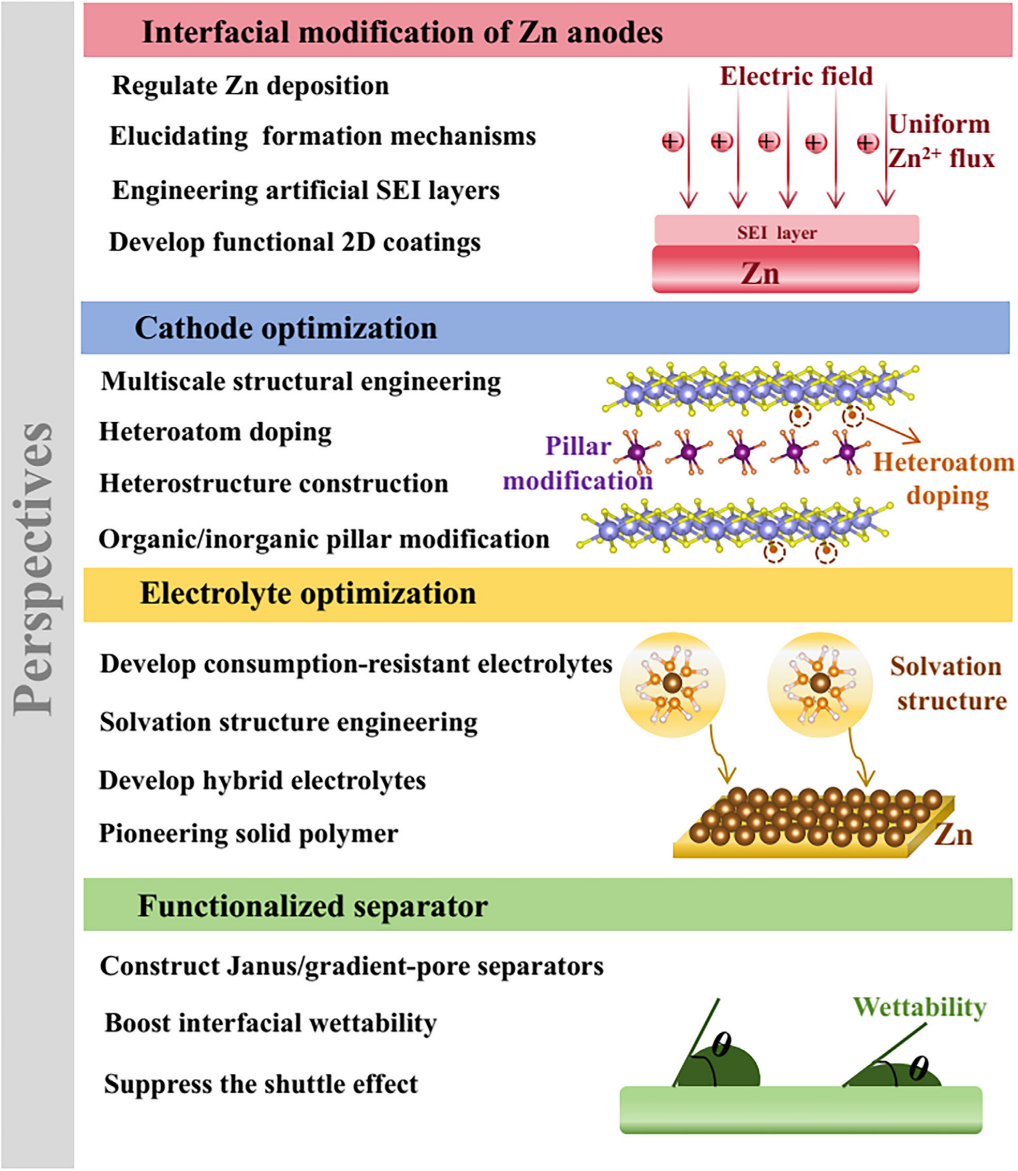
Perspectives for future fundamental research of AZIBs.

This review comprehensively overviews recent progress in representative 2D materials and conducts a systematic analysis of their multifunctional applications in AZIBs. We elaborate on pristine 2D materials, 2D‐derived configurations, and cutting‐edge interfacial engineering methodologies, emphasizing innovative design strategies and the indispensable role of these materials in the fabrication of high‐performance electrodes and key AZIB components ranging from anodes and cathodes to electrolytes and separators (Figure 18). Despite this progress, practical application remains hindered by challenges such as sluggish reaction kinetics, low discharge voltage, substantial polarization, and insufficient cycling stability. To address these issues, various strategies have been implemented to improve electrochemical performance, including electrode modification, electrolyte optimization, incorporation of redox mediators, and innovative battery designs. While notable progress has been made, the field remains in its early stages. In light of the limitations inherent in current approaches, we propose several prospective research directions to steer future development.

### 5.1 Interfacial Modification of Zn Anode

2D material coatings serve as a viable interfacial modification strategy to protect Zn anodes by isolating electrodes from corrosive electrolytes. Nevertheless, mainstream MXene, graphene, and LDH‐based coatings suffer from poor long‐term stability, unsatisfactory large‐scale uniformity, and limited electrolyte compatibility. Future advances require multifunctional interfacial design: optimizing 2D coatings with ion‐selective and electrically insulating features, and constructing composite interfaces to boost mechanical durability and ion transport. Integrating hydrophobic modification, Zn^2+^‐philic sites, and self‐healing functions into artificial interphases, alongside lightweight 3D host structures, is critical to achieving dendrite‐free, high‐stability Zn anodes.

### 5.2 Cathode Optimization

Future research on AZIB cathodes should focus on developing multifunctional composite materials that integrate high specific capacity, high working voltage, superior rate capability, and ultralong cycling stability. Vanadium‐ and manganese‐based cathode oxides suffer from inherent drawbacks, including active material dissolution, low electrical conductivity, and sluggish reaction kinetics, which can be effectively mitigated via multiscale structural engineering. Heteroatom doping, organic/inorganic pillar modification, and heterostructure construction can exert synergistic effects to optimize electrochemical behaviors. In particular, high‐entropy engineering and precise defect regulation (e.g., oxygen vacancy modulation) simultaneously stabilize the crystal structure and accelerate Zn^2+^ diffusion. Furthermore, in situ/operando characterizations of emerging 2D cathodes (e.g., transition metal dichalcogenides and phosphates) can deepen the understanding of Zn^2+^ storage mechanisms, guiding the rational design of advanced cathode materials. Overall, future cathode design aims to synergistically boost energy and power density while maintaining excellent long‐term capacity retention.

### 5.3 Electrolyte Optimization

Electrolyte optimization is central to advancing ZIBs. A primary challenge lies not only in dendrite suppression but more critically in mitigating capacity fade caused by electrolyte depletion. Future electrolyte development for ZIBs should prioritize three key strategies. First, molecular‐level design of novel salts and solvent systems is crucial to suppress water activity and stabilize the Zn anode interface. Second, smart electrolytes incorporating functional additives should be developed to autonomously form robust Zn^2+^‐conductive interphases. Third, research must advance solid‐state systems that simultaneously achieve high ionic conductivity and excellent interfacial contact while maintaining mechanical robustness. These approaches will collectively address critical challenges in interfacial stability and device safety.

### 5.4 Functionalized Separator

Separator functionalization plays a vital role in realizing ion‐selective transport and physically inhibiting dendrite penetration toward stable AZIBs. Future research should prioritize the design of advanced separators with Janus asymmetric architectures or gradient pore structures. Specifically, the anode‐facing surface can be decorated with negatively charged or functional‐group‐enriched 2D materials such as MXene and rGO to homogenize Zn^2+^ flux and repel harmful anions. In contrast, the cathode‐facing side can be rationally engineered to improve electrolyte wettability and ionic conductivity. Benefiting from such asymmetric structural design, the modified separators can effectively restrain zinc dendrite proliferation and alleviate the shuttle effect of dissolved cathode active substances, thereby substantially boosting the overall electrochemical performance and long‐term cycling durability of AZIBs.

## Author Contributions

**Shuge Dai**: Writing – review and editing, Writing – original draft. **Ye Wang**: data curation, formal analysis. **Chenke Yang**: data curation, formal analysis. **Gaopan Liu**: formal analysis, data curation. **Yuen Hong Tsang**: writing – review and editing, supervision. **Yunrui Jiang**: writing – review and editing, formal analysis. **Jianbin Zhou**: writing – review and editing, formal analysis. **Longhui Zeng**: writing – review and editing, supervision.

## Conflicts of Interest

The authors declare no conflicts of interest.

## Data Availability

The data that support the findings of this study are available from the corresponding author upon reasonable request.
